# Metformin acts in the gut and induces gut-liver crosstalk

**DOI:** 10.1073/pnas.2211933120

**Published:** 2023-01-19

**Authors:** Natália Tobar, Guilherme Z. Rocha, Andrey Santos, Dioze Guadagnini, Heloísa B. Assalin, Juliana A. Camargo, Any E. S. S. Gonçalves, Flavia R. Pallis, Alexandre G. Oliveira, Silvana A. Rocco, Raphael M. Neto, Irene Layane de Sousa, Marcos R. Alborghetti, Maurício L. Sforça, Patrícia B. Rodrigues, Raissa G. Ludwig, Emerielle C. Vanzela, Sergio Q. Brunetto, Patrícia A. Boer, José A. R. Gontijo, Bruno Geloneze, Carla R. O. Carvalho, Patricia O. Prada, Franco Folli, Rui Curi, Marcelo A. Mori, Marco A. R. Vinolo, Celso D. Ramos, Kleber G. Franchini, Claudio F. Tormena, Mario J. A. Saad

**Affiliations:** ^a^Department of Internal Medicine, School of Medical Sciences, University of Campinas, Campinas, 13083-887 Brazil; ^b^Department of Physical Education, São Paulo State University, Rio Claro, 13506-900 Brazil; ^c^Brazilian Biosciences National Laboratory and Brazilian Center for Research in Energy and Materials, Campinas, 13083-100 Brazil; ^d^Department of Genetics, Evolution, Microbiology and Immunology, Institute of Biology, University of Campinas, Campinas, 13083-970 Brazil; ^e^Department of Biochemistry and Tissue Biology, Institute of Biology, University of Campinas, Campinas, São Paulo, 13083-970; ^f^Department of Structural and Functional Biology, University of Campinas, Campinas, 13083-865 Brazil; ^g^Biomedical Engineering Center, University of Campinas, Campinas, 13083-881 Brazil; ^h^Institute Biomedical Sciences, University of Sao Paulo-Department of Physiology and Biophysics, São Paulo, 05508-000 Brazil; ^i^School of Applied Sciences, University of Campinas, Limeira, 13484-3501 Brazil; ^j^Butantan Institute-São Paulo, 05503-900 Brazil; ^k^Division of Nuclear Medicine, Department of Radiology, School of Medical Sciences, University of Campinas, Campinas, 13083-888 Brazil; ^l^Institute of Chemistry, University of Campinas, Campinas, 13083-970 Brazil

**Keywords:** Metformin, Diabetes, gut-liver crosstalk, glucose metabolism

## Abstract

Metformin is the most prescribed drug for DM2, but its site and mechanism of action are still not well established. Here, we investigated the effects of metformin on basolateral intestinal glucose uptake (BIGU), and its consequences on hepatic glucose production (HGP). In diabetic patients and mice, the primary site of metformin action was the gut, increasing BIGU, evaluated through PET-CT. In mice and CaCo2 cells, this increase in BIGU resulted from an increase in GLUT1 and GLUT2, secondary to ATF4 and AMPK. In hyperglycemia, metformin increased the lactate (reducing pH and bicarbonate in portal vein) and acetate production in the gut, modulating liver pyruvate carboxylase, MPC1/2, and FBP1, establishing a gut-liver crosstalk that reduces HGP. In normoglycemia, metformin-induced increases in BIGU is accompanied by hypoglycemia in the portal vein, generating a counter-regulatory mechanism that avoids reductions or even increases HGP. In summary, metformin increases BIGU and through gut-liver crosstalk influences HGP.

## Significance

The site and mechanisms of action of metformin are incompletely understood. Here, we show that in hyperglycemic conditions, metformin increases basolateral intestinal glucose uptake (BIGU), and its metabolites produced in the gut, through the portal vein reach the liver and reduce hepatic glucose production (HGP). In normoglycemic conditions, metformin increases modestly BIGU, inducing hypoglycemia in the portal vein, and in response there will be unincreased HGP. Although our data do not exclude a direct action of metformin in the liver, they indicate that the first site of metformin action is the gut, and through gut-portal vein-liver crosstalk, it may have a role in the control of HGP, integrating the sites and the mechanisms of metformin action.

Metformin is the most prescribed drug for treating type 2 diabetes (DM2), but its mechanism of action is still not fully understood (1–3). The most investigated site of action of metformin is the liver, where it may reduce hepatic glucose production (HGP) (1). However, this is not uniformly observed (4, 5), with data showing an increase in glucose utilization/effectiveness associated with increased HGP.

Some studies demonstrate that the metabolic benefit of metformin might be due to actions in the intestine (6–8). Recent data have shown that metformin modulates the gut microbiota without a well-characterized role in its antihyperglycemic effect (8, 9). Previous data showed that metformin concentrations are higher in the gut than in the blood (10–12), indicating that metformin action in the intestine may be relevant. In addition, intravenous administration (IV) of metformin is less effective than oral administration in reducing glucose levels (13, 14). Recent data demonstrated that oral administration of delayed-release metformin, with a low absorption rate, has similar effectiveness as standard release formulation in reducing fasting plasma glucose in DM2 (15, 16).

In the gut, metformin promotes glucose uptake through the basal side as demonstrated using imaging with positron emission tomography-computed tomography (PET/CT) (17). However, no previous study has evaluated the quantitative importance of the gastrointestinal tract in the antihyperglycemic effect of metformin, and if there is crosstalk between the intestine and the liver. The present study investigated how metformin increases basolateral intestinal glucose utilization (BIGU) and its consequences on HGP.

## Results

### Metformin Induces BIGU in Diabetic Patients.

**Fig. 1.**
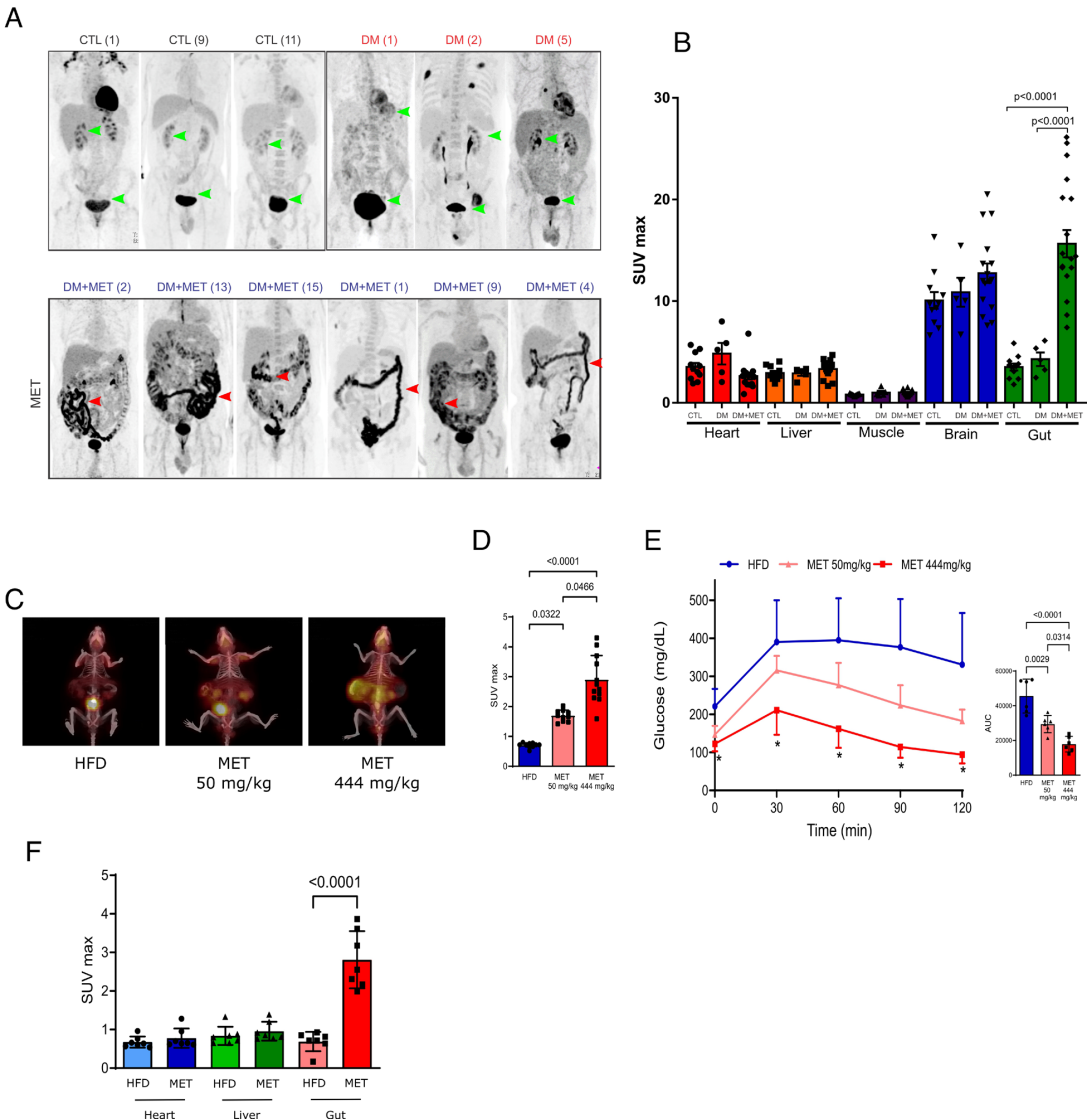
Metformin increases basolateral intestinal glucose uptake and utilization (BIGU) in diabetic patients and in obese mice and improves glucose tolerance in these mice. (*A*) Representative images of whole-body ^18^F-FDG PET/CT scanning from control individuals (CTL, n = 11) diabetic patients (DM, n = 5) and diabetic patients in use of metformin (DM+MET, n = 16). ^18^F-FDG PET/CT uptake is color-coded, and areas of increased signal exhibit black color. (*B*) ^18^F-FDG biodistribution analysis (SUVmax) in different tissues from CTL, DM and DM+MET diabetic patients. (*C*) Representative images of whole-body ^18^F-FDG PET/CT scanning from mice on HFD and treated with vehicle or metformin (MET) with low (50 mg/kg) and high (444 mg/kg) dose for 10 d and 2 h before the PET/CT. ^18^F-FDG PET/CT uptake is color coded, and areas of increased signal exhibit red-orange color. (*D*) ^18^F-FDG biodistribution analysis (SUVmax) in the intestine from mice treated with vehicle (HFD) or treated with metformin (MET) at a low and high dose, respectively. (*E*) Blood glucose levels from HFD and MET treated mice during a glucose tolerance test (GTT). (*F*) ^18^F-FDG biodistribution analysis (SUVmax) in different tissues from HFD and MET mice (50 mg/kg/day for 10 d). All tests performed were one-way ANOVA with Bonferroni’s post-test.

We used the archives of the Nuclear Medicine Service of our University Hospital to identify diabetic patients submitted to PET/CT on metformin and without metformin. Our search showed that diabetic patients on metformin (n = 16) submitted to PET/CT scanning presented an increase in 2-deoxy-2-[18F]fluoro-D-glucose (18F-FDG) uptake all over the intestine, including jejunum, ileum, and colon, compared to diabetic patients without metformin (n = 5) and to controls (n = 11) (Fig. 1*A*) (*SI Appendix*, Table S1). To quantify the relative contribution of the intestine to whole-body glucose disposal, we performed biodistribution analysis using 18F-FDG in both groups of patients and also of controls. The intestine exhibited the highest glucose uptake rate and became a significant tissue for glucose disposal after treatment with metformin (Fig. 1*B*).

### Metformin Increases BIGU in Mice on a High-Fat Diet (HFD).

To investigate more deeply the mechanism by which metformin enhances intestinal glucose uptake and utilization, we first examined whether the effect observed in humans is also reproducible in mice. We investigated whether different doses of metformin for 10 d improve glucose tolerance and increase BIGU. The results showed that the improvement in glucose tolerance presents a dose–response curve, with the maximal effect observed after 200 to 444 mg/kg (*SI Appendix,* Fig. S1*A*). Although 50 mg/kg of metformin (MET) significantly increased BIGU, a more pronounced increase in BIGU was observed with 444 mg/kg in parallel to a more efficient improvement in glucose tolerance (Fig. 1 *C*–*E*). The results also showed that the increase in glucose uptake after 50 mg/kg of metformin was only observed in the intestine (Fig. 1*F*). We present most of our data with a metformin dose of 50 mg/kg/day in mice because this dose gives plasma metformin concentration similar to humans using 2 g/day (18). However, we also presented the results with the highest dose in supplemental figures, because the dose of 444 mg/kg of metformin gives the highest response in the glucose tolerance test (*SI Appendix,* Fig. S1) and in BIGU in our animal model.

We also determined metformin concentration on the 10th day after 50 mg/kg/day or 444 mg/kg/day 2 h after the last gavage, in the portal vein, in the ileum and liver, and after 8 h in the colon (*SI Appendix* Table S2). The results showed higher concentrations of the drug in the ileum and colon, independent of the dose used. However, a dose-dependent increase in the levels was observed in the portal vein and the liver. Portal vein levels and tissue levels of metformin obtained after 50 mg/kg/day, 2 h after the last gavage, are very similar to previous data which are in the therapeutic range (15, 18).

### Mechanism of Increased BIGU.

**Fig. 2.**
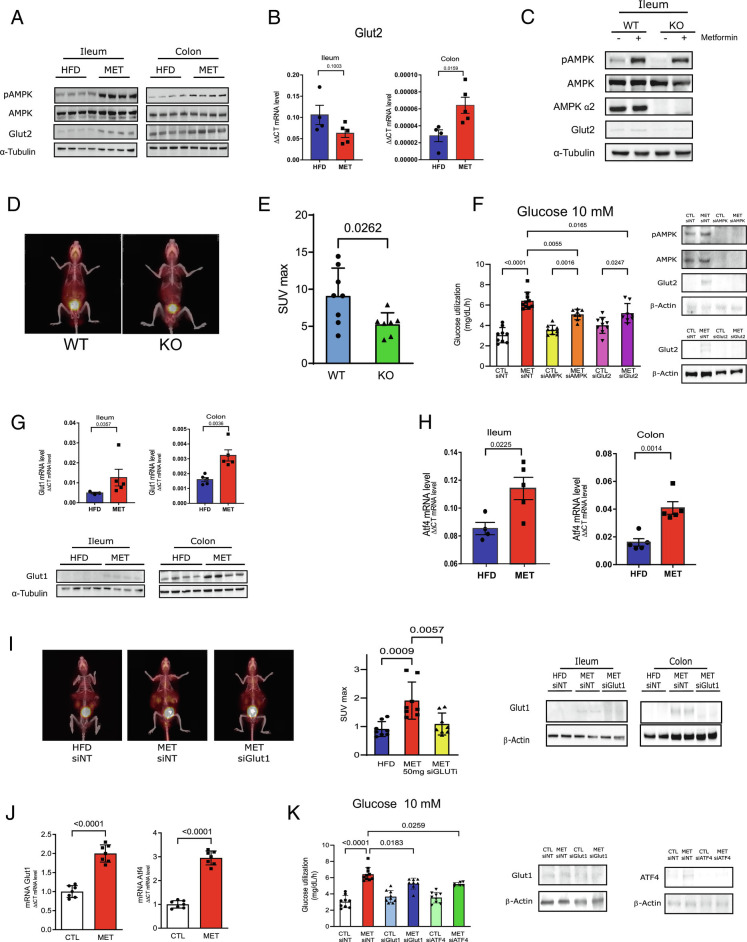
Metformin induces AMPK phoshorylation and modulates GLUT2 and GLUT1 in ileum and colon. (*A*) Protein phosphorylation and expression of AMPK and protein expression of Glut2 in ileum and colon from HFD and MET-treated mice (50 mg/kg for 10 d). (*B*) Glut-2 mRNA expression in ileum and colon from HFD and MET mice (50 mg/kg for 10 d). (*C*) Protein phosphorylation and expression of AMPK and expression of Glut2 in ileum from wild type (WT) and AMPK knock-out (KO) littermates mice treated or not with metformin (MET) (50 mg/kg for 10 d). (*D*) Representative images of whole-body ^18^F-FDG PET/CT scanning from mice and ^18^F-FDG biodistribution analysis (SUVmax) in the intestine from AMPK wild type (WT) and knock-out (KO) mice treated with metformin and (*E*) SUVmax quantification in the intestine. (Student’s *t* test, *P* < 0.05, n = 5). (*F*) Glucose uptake at 10 mM glucose concentrations in CaCo2 cells after the incubation with metformin (MET 1 mM to 16 h) in cells pre-treated with siRNA of AMPK, GLUT2 and non-targeting, as control (siNT) (one-way ANOVA and Bonferroni’s test, *P* < 0.05). (*G*) Glut-1 mRNA and protein expression in ileum and colon from HFD and MET mice (50 mg/kg for 10 d). (*H*) ATF-4 mRNA expression in ileum and colon from HFD and MET mice (50 mg/kg for 10 d) (n = 6). (*I*) Representative images of whole-body ^18^F-FDG PET/CT scanning from mice and ^18^F-FDG biodistribution analysis (SUVmax) in the intestine from mice treated with non-targeting siRNA or Glut1siRNA and with metformin (50 mg/kg for 10 d) and expression of Glut1 in ileum and colon (ANOVA, Bonferroni post test, *P* < 0.05, n = 5). (*J*) Glut-1 and ATF4 mRNA expressions in CaCo2 cells. (*K*) Glucose uptake at 10 mM glucose concentrations in CaCo2 cells after the incubation with metformin (MET 1mM to 16h) and siRNA-mediated knockdown of GLUT1, ATF4 and non-targeting, as control (siNT) and expression of Glut1 and Atf4 in CaCo2 cells (one-way ANOVA and Bonferroni’s test.)

Previous data showed that metformin actions inducing AMPK phosphorylation in the duodenum (19) are important for its antihyperglycemic effect. Our data show that 50 mg/kg of metformin induces AMPK phosphorylation in the ileum and colon of HFD mice, and in parallel induces an increase in GLUT2 protein expression (Fig. 2*A*), the primary glucose transporter in the basolateral side, in ileum and colon (20). We then investigated the effect of metformin on the expression and distribution of GLUT2. The results showed that metformin increased GLUT2 mRNA in the colon but not in the ileum and increased tissue protein levels in whole tissue extracts and plasma membrane in both tissues (Fig. 2*B* and *SI Appendix,* Fig. S2*A*). In the ileum of HFD mice, GLUT2 also co-localized in the nucleus (*SI Appendix,* Fig. S2*B*), and metformin treatment reduced GLUT2 in the nucleus, increasing this glucose transporter in the plasma membrane. This distribution may explain the lower level of this protein in the ileum of HFD mice because the concentration of cytoplasmic GLUT2 is probably diluted by the vast array of cytoplasmic protein in whole-tissue extracts pre-treatment. In addition, by using immunofluorescence, we observed that obese mice presented higher levels of GLUT2 in the apical side of the intestine. After metformin treatment, there was an evident translocation of GLUT2 to the basolateral side of the intestinal epithelium of the ileum and colon (*SI Appendix,* Fig. S2*C*–*D*).

We subsequently investigated whether inhibition of AMPKα2 can blunt this effect of metformin on BIGU. Although AMPKα2 knockout (KO) mice have only a partial reduction in AMPK activation, these animals are the only AMPK KO mice without changes in the epithelial architecture of the intestine (21). In the ileum, there was a moderate reduction in metformin-induced AMPK phosphorylation in AMPKα2 KO mice, associated with a reduction in GLUT2 protein expression, and in parallel, there was also a significant decrease in metformin-induced BIGU in these mice (Fig. 2 *C*–*E*). These data indicate that partial reduction in AMPK and/or AMPKα2 deletion blunts the metformin effect on BIGU.

Next, we investigated whether inhibition of AMPK, in two different concentrations of glucose in the medium, can also blunt this effect of metformin in CaCo-2 cells. Metformin-induced glucose utilization at 5 mM glucose was independent of AMPK or GLUT2 (*SI Appendix,* Fig. S3*A*). At 10 mM glucose metformin increased glucose uptake, and siRNA of AMPK dramatically reduces the expression of GLUT2 and metformin-induced glucose uptake (Fig. 2*F*). As expected, siRNA of GLUT2 reduces the expression of GLUT2 and metformin-induced glucose uptake (Fig. 2*F*). These data suggest that metformin independent of the glucose concentration, increase glucose uptake in cells. However, only at 10 mmol glucose, the effect of metformin depends on AMPK and GLUT2.

Since at 5 mM glucose metformin increases glucose uptake independent of GLUT2, we decided to investigate the effect of metformin in other glucose transporters. Previous data showed that the fetal intestine expresses GLUT1 (20, 22). We investigated whether metformin could increase the expression of this glucose transporter in the ileum and colon. The results showed that metformin induces an increase in mRNA and protein levels of GLUT-1 in both tissues (Fig. 2*G* and *SI Appendix,* Fig. S3*B*). Previous data showed that metformin induces the integrated stress response through ATF4 (23), and that ATF4 promotes a transcriptional enhancement of the GLUT1 (24). We then investigated whether metformin could increase the expression of ATF4. The results showed that metformin increased ATF4 mRNA and protein in the ileum and colon (Fig. 2*H* and *SI Appendix,* Fig. S3*C*), suggesting that ATF4 may have a role in the effect of metformin on GLUT1 expression.

PET/CT scanning of HFD mice treated with an administration of siGLUT1 blunted metformin-induced intestinal 18F-FDG uptake (Fig. 2*I*), suggesting that GLUT1 mediates metformin-induced BIGU. In CaCo-2 cells, metformin-induced GLUT1 and ATF4 expression (Fig. 2*J*) and glucose utilization in different concentrations of glucose (Fig. 2*K* and *SI Appendix,* Fig. S3*D*). Our data also showed that at 5 mmol/L and at 10 mmol/L the knockdown of ATF4 or GLUT1 reduced metformin-induced glucose uptake in CaCo-2 cells (Fig. 2*K* and *SI Appendix,* Fig. S3*D*), reinforcing the importance of ATF4 and GLUT1 in metformin-induced glucose uptake in the intestine.

### Metabolomics and Glucose Utilization.

**Fig. 3.**
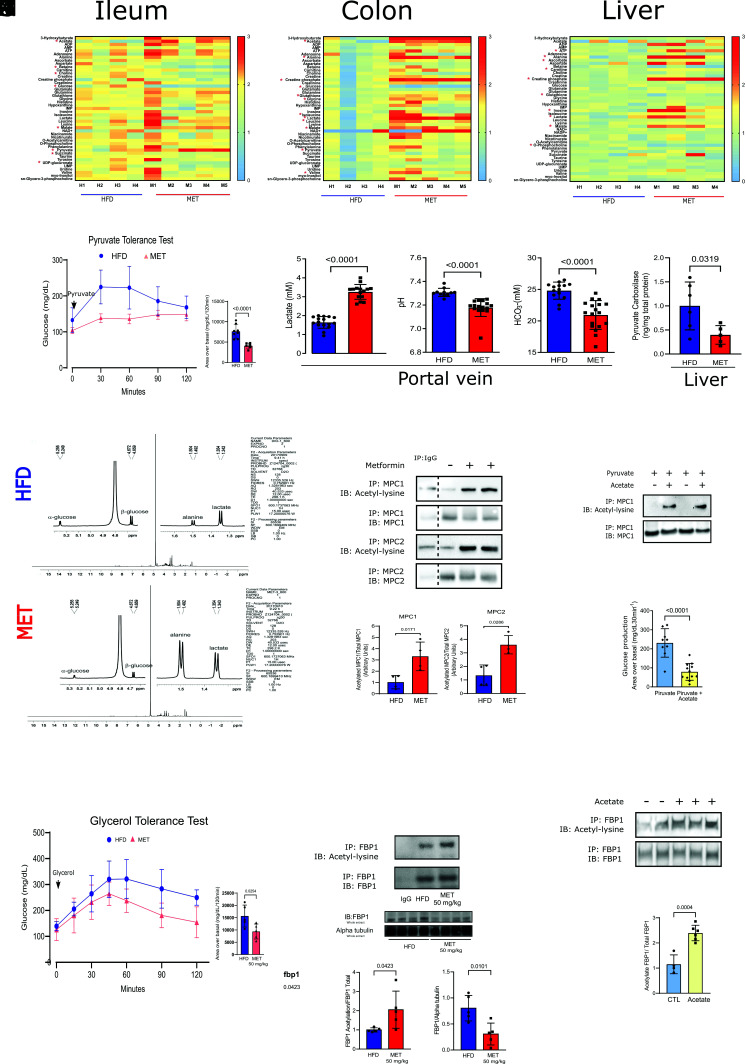
Metformin increases glycolysis in ileum and colon and reduces HGP in animals on HFD (*A*–*C*) Metabolomic analysis of ileum, colon and liver from the HFD and MET mice treated (50 mg/kg/day) for 10 d (one-way ANOVA and Bonferroni’s test, *P < 0.05, n = 4 to 5) (*D*) PTT curve and area under curve of metformin treated mice (50 mg/kg/day for 10 d) (n = 5). (*E*) Lactate, pH and bicarbonate levels in blood from the portal vein of HFD and MET Wistar rats. (*F*) Pyruvate carboxylase levels in liver from HFD and MET treated Wistar rats. (*G*) (1H) NMR spectra acquired at 600 MHz in D_2_O. (*H*) Acetylation of MPC1/2 in the liver of HFD and MET treated C57BL6/J mice (n = 5). (*I*) Acetylation of MPC1 in HuH7 cells treated with pyruvate and acetate. (*J*) Glucose production of Huh7 cells treated with pyruvate and acetate. (*K*) Glycerol tolerance test curve and area under curve of metformin treated mice (50 mg/kg/day for 10 d) (n = 5). (*L*) Acetylation and tissue protein levels of FBP1 in the liver of HFD and MET treated C57BL6/J mice (n = 5). (*M*) Acetylation of FBP1 in HuH7 cells treated with acetate.

To better understand the fate of glucose metabolism in the ileum, colon, and liver, we performed metabolomics of these tissues from HFD mice treated or not with metformin (50 mg/kg/day for 10 d). In the ileum, colon, and liver, there was an increase in amino acids and metabolites which are substrates of gluconeogenesis, indicating inhibition of this pathway in the gut and liver (Fig. 3 *A*–*C*). In accordance, this low dose of metformin reduced HGP evaluated through the pyruvate tolerance test (PTT) (Fig. 3*D*). It is interesting that in metformin-treated mice, despite an increase in glucose uptake in the gut, there was also a decrease in glucose in the ileum and colon and an increase in pyruvate and lactate suggesting glucose metabolism through the glycolytic pathway in these tissues (Fig. 3 *A* and *B*). However, in liver, there was no decrease in glucose levels after metformin treatment and only a mild increase in lactate. These data might suggest a tissue-specific effect of metformin but we cannot exclude the possibility that the increase in liver lactate after metformin may reflect an increase in lactate coming from the gut. The increase in malate in the three tissues is not easy to explain, but may reflect an increase or inversion of the malate-aspartate shuttle, and may also indicate that metformin increases lactate oxidation, as previously described (25, 26). Metformin induced an increase in acetate in ileum and colon (Fig. 3  *A* and *B*), which may be secondary to hyperactive glucose metabolism as previously demonstrated and/or increased deacetylation.

In mice treated with higher doses of metformin, we observed changes in the same direction induced by lower doses (*SI Appendix,* Fig. S4 *A*–*C*). It is noteworthy that independent of the dose, metformin-induced glucose metabolism in the glycolytic pathway in the ileum and colon, increasing lactate concentration (*SI Appendix,* Fig. S4 *A* and *B*). This lactate is predominantly L-lactate, evaluated through RMN spectroscopy, excluding an overproduction of lactate from microbiota (of which the lactate is mainly D-lactate) (*SI Appendix,* Fig. S4*D*). Metformin also induced an increase in acetate in Ileum and colon (*SI Appendix,* Fig. S4 *A* and *B*). Similar to 50 mg/kg/day, the high dose of metformin also induced only a mild increase in acetate and lactate in the liver suggesting an effect of metformin in the liver and/or that these substrates came from the ileum and colon, reaching the liver through the portal vein. There was an increase in precursors of gluconeogenesis in the liver, indicating a block of this pathway (*SI Appendix,* Fig. S4*C*). The increase in glucose-6-phosphate (G-6-P), glucose-1-phosphate (G-1-P), and uridine diphosphate glucose with the high doses suggest that the glycogenesis pathway is activated in the liver (*SI Appendix,* Fig. S4*C*). This is an expected result after 10 d of high doses of metformin treatment since previous data showed that the improvement in diabetes control and insulin resistance increases the glycogenic pathway (27).

### Gut-Liver Crosstalk.

The PTT confirmed that chronic metformin also reduced HGP in HFD mice in a dose-dependent manner (Fig. 3*D* and *SI Appendix,* Fig. S4*E*). Based on our metabolomics study, we investigated whether some of the metabolites from the intestine, modulated by metformin, could establish crosstalk with the HGP pathway. We came up with two suggestions: a modulation of pyruvate carboxylase (PC), a gluconeogenic enzyme, by pH and bicarbonate (28) secondary to the increase in lactate, and possible modulation of pyruvate carrier transport (MPC1/2) by acetylation (29–32), a consequence of increased acetate.

Our data showed that the lactate produced in the ileum and colon is delivered into mesenteric circulation and can reduce the pH and bicarbonate levels in the portal vein (Fig. 3*E*). However, after 12 h of metformin administration, the peripheral levels of lactate decreased to normal levels (*SI Appendix,* Fig. S4*F*). The increase in lactate oxidation induced by metformin (33) may have contributed to this result. Interestingly, PC is very sensitive to changes in pH, and reducing pH can dramatically reduce the activity of this enzyme (28). Moreover, the reduction in bicarbonate may also refrain gluconeogenesis because it is necessary to convert pyruvate into oxaloacetate. In accordance, metformin treatment reduced bicarbonate levels and pH in the portal vein and, in parallel, PC tissue protein levels in the liver of HFD mice (Fig. 3*F*). To show that metformin reduces pyruvate flux through gluconeogenesis in mice, we used a labeled 13C-pyruvate injection in the portal vein and investigated the effect of metformin on the flux of pyruvate through gluconeogenesis by 1H and 13C NMR spectroscopy. The results showed that in the liver of HFD mice without metformin, the detection of labeled 13C-pyruvate is very low or absent. However, signals from glucose, lactate, and alanine were detected in a similar amount, indicating that the injected pyruvate is quickly converted to these metabolites. In contrast, in animals treated with metformin, a signal from labeled 13C-pyruvate is also not observed, but lactate and alanine signals predominate over glucose (Fig. 3*G*),. These data suggest that metformin reduced gluconeogenic flux through PC.

In HFD Wistar rats treated with metformin plus NaHCO3, there was an attenuation of the metformin effect on HGP in parallel to an increase in portal pH (*SI Appendix,* Fig. S5*A*), and the acidification of the portal vein with NH4Cl reduced HGP (*SI Appendix,* Fig. S5*B*). Moreover, we observed a negative and significant correlation between portal lactate levels and HGP (*SI Appendix,* Fig. S5*C*) and a positive correlation between pH or bicarbonate with HGP (*SI Appendix*, Fig. S5 *D* and *E*). Moreover, we also infused pyruvate, in the PTT, diluted in phosphate buffer with different pHs, and the results showed that pyruvate diluted at pH 7.1 induced a blunted glucose curve compared to pH 7.9 (*SI Appendix,* Fig. S5*F*). In Huh7 cells, we also showed that decreasing the pH from 7.4 to 7.2 reduced HGP generated by pyruvate, in parallel to a reduction in PC levels (*SI Appendix,* Fig. S5 *G* and *H*).

Subsequently, we investigated if metformin treatment can induce acetylation in MPC1/2 in the liver. The results showed that treatment with metformin could increase acetylation and decrease MPC1/2 protein levels (Fig. 3*H*). An increase in acetylation may be a consequence of the deactivation of deacetylases and/or an increase in acetyl-CoA and acetylases. In this regard, previous data showed that metformin could reduce SIRT3, which is a critical mitochondrial deacetylase(30). However, since our data showed a marked increase in acetate induced by metformin, we would like to emphasize that acetate-derived acetyl groups could contribute to the cellular acetylation reactions (34). Moreover, the dependence on acetate to supply, in different cellular compartments, Acetyl-CoA pools have been identified in multiple cell types (35, 36). We subsequently investigated this possibility in mice and cells. We treated HFD mice with low acetate doses and observed a decrease in HGP during PTT (*SI Appendix,* Fig. S5*I*). Accordingly, by treating Huh7 cells with acetate, there was an increase in acetylation and degradation of this carrier, associated with reduced HGP in these cells after pyruvate (Fig. 3 *I* and *J*).

The crosstalk in PC and MPC1/2 induced by metformin may contribute to explaining the reduction in HGP from pyruvate but our data also showed that metformin blunts glycerol-induced HGP (Fig. 3*K* and *SI Appendix,* Fig. S6*A*). In this regard, we investigate whether other proteins from the gluconeogenic pathway may also be acetylated. Previous data showed that Fructose 1,6-bisphosphatase (FBP1) (5, 37), a critical enzyme of gluconeogenesis, in addition to being inhibited by AMP, can also be acetylated which is accompanied by reduced activity (38). We then investigated whether metformin treatment can induce acetylation of FBP1. The results showed that metformin can increase acetylation, corrected by FBP1 protein level (that showed a marked decrease in the liver) (see Fig. 3*L*). Previous data showed that metformin by itself does not modify the activity of FBP1 (39). We then investigated whether acetate can induce FBP1 acetylation in cells. By treating HuH7 cells with acetate, there was an increase in acetylation of this enzyme (corrected by FBP1 protein level that was reduced), associated with reduced HGP in these cells after pyruvate (Fig. 3*M*).

### Metformin Effect under Normoglycemia.

**Fig. 4.**
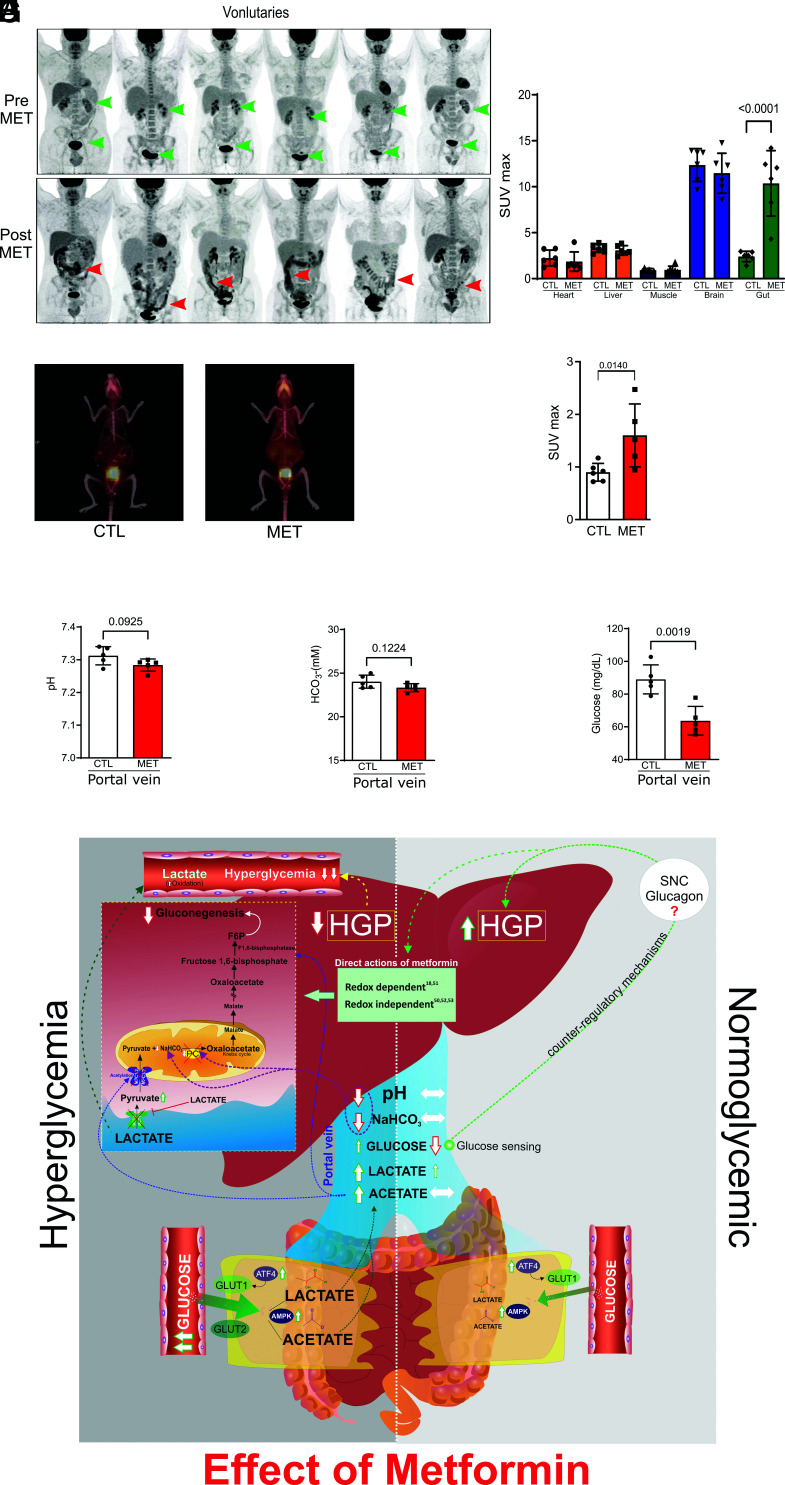
Metformin induces moderate increase BIGU in control individuals and normal glucose-tolerant rodents and does not reduce HGP in the liver of these rodents. (*A*) Representative images of whole-body 18F-FDG PET/CT scanning from the same healthy subjects before (PRE) and after metformin (2 g/day for 5 d) (POST). 18F-FDG PET/CT uptake is color coded, and areas of increased signal exhibit black color. (*B*) 18F-FDG biodistribution analysis (SUVmax) in different tissues from CTL and MET subjects (one-way ANOVA and Bonferroni’s test, *P* < 0.001, n = 6). (*C*) Representative images of whole-body ^18^F-FDG PET/CT scanning from mice on control chow mice treated with vehicle or metformin (MET 50 mg/kg/day for 10 d) and for 2 h before the PET/CT, ^18^F-FDG PET/CT uptake is color coded, and areas of increased signal exhibit red-orange color. ^18^F-FDG PET/CT images from lean mice (CTL) and treated with vehicle or metformin (MET). (*D*) SUVmax in the intestine of CTL and MET treated mice (50 mg/kg/day for 10 d) (one-way ANOVA and Bonferroni’s test, *P* < 0.05, n = 5 to 6). (*E* and *F*) pH and bicarbonate levels in blood from the portal vein of CTL and MET (250 mg/kg/10 d) treated Wistar rats. (*G*) Glucose levels in CTL and MET treated (250 mg/kg/day for 10 d) in lean Wistar rats (Student’s *t* test, *P* < 0.05, n = 5). (*H*) Schematic representation of metformin actions in conditions of hyperglycemia and normoglycemia. In hyperglycemic conditions, metformin induces GLUT1 and GLUT2 in the colon and ileum, where both these glucose transporters are expected to promote BIGU. In enterocytes, the glucose is metabolized to lactate, which will decrease pH and NaHCO_3_ in the portal vein and decrease gluconeogenesis via PC inhibition. In parallel, an increase in acetate production in the gut will induce acetylation and inhibit MPC1/2, leading to cytosolic accumulation of pyruvate, which in turn prevents the uptake of extracellular lactate through MCT1. Acetylation also blocks FBP1. These mechanisms demonstrate that metformin establishes a crosstalk between gut and liver to reduce gluconeogenesis in hyperglycemic conditions. In normoglycemic conditions, metformin induces GLUT1 and 2 expressions, but considering the Km of the glucose transporters, GLUT1 is expected to be preferentially used to modestly increase BIGU. In this condition, the increase in lactate will be discreet, and no alteration in acid–base equilibrium in the portal vein will be observed. The moderate increase in BIGU will induce hypoglycemia in the portal vein, which can induce portal glucose sensing and a possible counter-regulatory response that will avoid a decrease in HGP or even increase it. It is important to mention that other actions of metformin directly in the liver can synergize with the demonstrated gut-liver crosstalk to reduce HGP. However, in conditions of normoglycemia, the counter-regulatory mechanisms may overcome these direct actions.

In conditions of normoglycemia or only mild hyperglycemia, metformin does not reduce HGP (4, 5). We then investigated the effect of metformin on BIGU in control individuals and BIGU and HGP in rats with standard glucose tolerance. Metformin induced a mild to moderate increase in BIGU in control individuals (Fig. 4 *A* and *B*) and in control mice (Fig. 4 *C* and *D*). In lean rodents, metformin does not change acid–base equilibrium in the portal vein (Fig. 4 *E* and *F*), nor lactate and acetate in the gut, without changing gluconeogenesis precursors in the liver (*SI Appendix,* Fig. S7 *A*–*C*), indicating no suppression of HGP. However, there was an apparent decrease in plasma glucose levels in the portal vein to hypoglycemic levels (Fig. 4*G*), which might induce glucose sensing in this vein, responding by increasing HGP. In accordance, in lean mice treated with metformin glycerol did not change but pyruvate increased HGP (*SI Appendix,* Fig. S7 *D* and *E*). In this regard, we can suggest that in conditions of normoglycemia or mild hyperglycemia metformin increases BIGU mildly, and the hypoglycemia in the portal vein, through glucose sensing, may overcome a direct or indirect effect of metformin in the liver, increasing HGP as described (40, 41).

## Discussion

The results of the present study showed that the main site of the glucose-lowering effect of metformin is the gut, and the initial glycemia and the amount of glucose metabolized by the gut contribute to determining HGP. In hyperglycemic conditions the marked increase in BIGU is accompanied by gut-liver crosstalk through metabolites that contribute to reducing HGP and, in normoglycemic conditions, the increase in BIGU induces hypoglycemia in the portal vein that generates a counter-regulatory response that might increase HGP.

Metformin-induced AMPK phosphorylation in the ileum and colon can have a role in increased BIGU, because in KO mice for AMPK2alpha and the genetic inhibition of AMPK in CaCo2 cells both showed a reduction in metformin-induced glucose uptake, in parallel to a reduction in GLUT2 expression. Additionally, metformin also induced GLUT1 expression, in parallel to an increase in ATF4, which certainly contribute to increase glucose uptake in the gut. The induction of two glucose transporters with different Km makes metformin action unique. In this regard, metformin-induced BIGU more pronounced in diabetic patients and HFD mice than in controls may also reflect the action of GLUT2, but the increase in GLUT1 is also important for metformin's effect in different concentrations of plasma glucose, indicating the relevance of the two glucose transporters. Metformin-induced BIGU may also explain its effect on improving glucose effectiveness without changing insulin sensitivity (42) or HGP (43).

In accordance with our data, robust evidence coming from different sources showed the importance of the intestine in the glucose-lowering effect of metformin. When small intestinal AMPK was knocked down in HFD-treated rats, the glucose-reducing effect of metformin was blunted (19). Very recently, Ma et al. demonstrated that low-dose metformin targets the lysosomal AMPK pathway through PEN2, but only intestine-specific knockout of Pen2 impairs the glucose-lowering effects of metformin (44).

Once in the gut, metformin-induces glucose utilization predominantly through the glycolytic pathway generating lactate and acetate (45, 46). Lactate reduces bicarbonate and pH in the portal vein, modulating an early step of gluconeogenesis in the liver probably at two different sites. First, the carboxylation of pyruvate by PC to oxalacetate requires bicarbonate; and second, PC is very sensitive to pH, and even mild decreases in pH markedly reduce its activity, suggesting that the reduction in bicarbonate and pH in portal vein induced by metformin may contribute to blunt HGP (28, 47, 48).

In parallel, there was an increase in MPC1/2 and FBP1 acetylation, which might involve acetylases and/or deacetylases. However, we cannot rule out the possibility that acetate can contribute to the induction of acetylation of MPC1/2 and FBP1 (30) The acetylation of MPC1/2 reduces its protein expression and activity (30, 32), reducing pyruvate transport to mitochondria (31), and impairing the initiation of gluconeogenesis. Although lactate transport into mitochondria depends on MCT1, recent data showed that the blockade of pyruvate imported into mitochondria prevents extracellular lactate uptake as efficiently as an MCT1 inhibitor (49). The acetylation of FBP1 also reduced its protein levels, explaining the effect of metformin reducing not only pyruvate-induced glucose production but also glycerol-induced glucose production. These data suggest that lactate and acetate generated in the intestine, can establish gut-liver crosstalk, reducing HGP and that acetylation might be an important post-translational modulator of gluconeogenesis, which deserves further investigation (Fig. 4*H*).

These findings do not exclude other direct actions of metformin on liver gluconeogenesis as previously described (18, 50–53). However, the metabolomics showed differences in glucose metabolism induced by metformin between the gut and liver that deserves consideration. The reduction in glucose levels is accompanied by an increase in pyruvate and lactate in the ileum and colon, but in the liver the increase in lactate is not accompanied by an increase in pyruvate or a decrease in glucose, suggesting a tissue-specific metabolic pathway regulation induced by metformin and/or that the increased lactate in the liver is coming from the gut (lactate was increased in portal vein), reinforcing the gut-liver crosstalk. In addition, our data also highlight the important role of the gut on the glucose-lowering action of metformin: a) the levels of metformin in the ileum and colon are much higher than in the liver, after gavage of lower or higher doses of the drug; b) HGP seems to be driven by BIGU, because in hyperglycemic conditions there is an increase in BIGU accompanied by a reduction in HGP, and in normoglycemic conditions, the mild increase in BIGU does not reduce HGP or even increase; c) confirming the importance of acidosis in the portal vein and the increase in acetate to control gluconeogenesis, the treatment with bicarbonate abolishes at least partially the effect of metformin on HGP and the infusion of a low dose of acetate blunts HGP in obese animals. Taken together these data indicate that a gut-liver crosstalk also has a role in the glucose-lowering action of metformin.

Our data also explain the paradoxical effect of metformin increasing HGP in individuals with normal glycemia (4, 5). In normoglycemic mice, metformin increases BIGU to a lesser extent than in diabetic mice but is sufficient to induce hypoglycemia in the portal vein. It does not decrease bicarbonate and pH in the portal vein, avoiding the gut-liver crosstalk that reduces HGP. It is possible that the hypoglycemia in the portal vein, through the portal vein sensing and portal-hepatic cycle (40, 41, 54), activates counter-regulatory mechanisms to increase HGP, opposing indirect (through gut-liver crosstalk) and/or direct actions metformin in the liver (18, 50–53) supposed to reduce HGP. We can suggest that the anatomical location of this glucose sensor in the portal vein and its physiological properties are very appropriate to prevent systemic hypoglycemia induced by metformin, which is very rare or nonexistent. In accordance, previous data showed that in non-diabetic individuals and individuals with recent-onset DM2 (4)  and also in prediabetes (55) metformin increases glucagon levels. Interestingly, our data showed in non-diabetic mice that pyruvate but not glycerol increased HGP. Since glucagon increases the activity of PC, PEPCK (56, 57), and MPC1 (58) we can suggest that glucagon is more efficient to stimulate gluconeogenesis starting from pyruvate than glycerol. Previous data also showed that glucagon is not able to increase HGP from glycerol (59, 60)*.*

The hypoglycemia in the portal vein induced by metformin can also be considered gut-liver crosstalk to drive HGP (Fig. 4*H*). Even though metformin might have direct actions in the liver modulating gluconeogenesis (18, 50–53), this effect might not be dominant because in conditions of normoglycemia it is supplanted by mild hypoglycemia in the portal vein, with the final effect being an increase in HGP.

In summary, our data show mechanisms of metformin action, integrating effects in the gut and the liver, without excluding the direct effect of the drug on liver enzymes. Metformin has unique actions, modulating BIGU and inducing metabolic gut-liver crosstalk, which modulates HGP in opposite directions depending on initial blood glucose levels.

## Research Design and Methods

### Materials.

#### Humans.

##### PET/CT.

Patients in the control group are under medication for their respective morbidities, but none has taken metformin. Patients that are in use of metformin are in use of medication for their respective diseases. ^18^F-FDG was injected in an arm vein of patients fasted for 6 h. The dose of ^18^F-FDG is calculated using the patient weight (kg) multiplied by 0.12 (mCi/kg), which then gives the dose of ^18^F-FDG in mCi. All participants in this study provided written informed consent. The present study was approved by CEP/FCM/UNICAMP through CAAE: 38906820.9.0000.5404.

### Animals.

Male Swiss, C57BL6/J and B6.129-Prkaa2tm1.1Vio/Orl (AMPK alpha2 KO) mice and Wistar rats were provided by the Multidisciplinary Center for Biological Investigation on Laboratory Animal Science—Unicamp (Campinas, Brazil). Metformin and routine reagents were purchased from Sigma Chemical Co. (St. Louis, MO). Animal procedures were performed according to the guidelines of the local animal care and use committee. The Ethics Committee of the University of Campinas approved all experiments (CEUA 4437-1 A and B). Eight-week-old male mice were maintained under specific pathogen-free conditions in a regimen of 12-h dark, 12-h light cycles, and room temperature of 21 °C. The animals were submitted to a HFD for 8 wk (HFD) (61) to induce obesity, insulin resistance, and glucose intolerance. Food and water were ad libitum.

## Metformin Administration Protocol

### Mice.

Metformin was diluted in the drinking water for 10 d and 2 h before all experiments. The drug was administered by gavage at a final dosage of 444 mg/kg (MET group). HFD group received only a vehicle solution (water). Low-dose metformin treatment (50 mg/kg) was administered by gavage.

### Rats.

Metformin was diluted in the drinking water for 10 d and 2 h before all experiments, and the drug was administered by gavage at a final dosage of 250 mg/kg (MET group). HFD group received only a vehicle solution (water).

### Assays.

Blood glucose was measured from the tail venous blood of all animals with a glucose analyzer. Lactate, pH, and HCO3- were measured from the portal vein of Wistar rats with a Blood gas analyzer—ABL800 FLEX—Radiometer.

### Tissue Extraction, Immunoprecipitation, and Protein Analysis by Immunoblotting.

After 12 h fasting, animals were anesthetized by intraperitoneal injection of Ketamine (100 mg/kg) and Xylazine (10 mg/kg). The abdominal cavity was opened 10 to 15 min later, i.e., as soon as anesthesia was assured by the loss of pedal and corneal reflexes. The portal vein and the aorta artery were exposed, and blood was taken in syringes specific to gasometry analysis. Then, the liver, colon, and the final third of ileum were extracted, washed, minced coarsely, and homogenized immediately in the extraction buffer. Experiments utilizing immunoprecipitation or immunoblotting were performed according to previously published work (61).

### Real-Time PCR.

Total RNA was obtained from the ileum and colon from both groups of mice according to the methods published previously (62) and CaCo2 cells according to the manufacturer’s protocol (RNeasy Mini Kit—QIAGEN). For tissue or cell samples, the first-strand cDNA was synthesized using SuperScript II reverse transcriptase as described in the manufacturer’s protocol (Invitrogen Corp.). Quantitative PCR was run to determine the expressions of GLUT1, GLUT2, and ATF4 in each tissue fraction and GLUT1 and ATF4 in CaCo2 cells. Real-time detection of amplification was performed in an QuantStudio™ Real-Time PCR Systems (Applied Biosystems) using TaqMan™ Gene Expression Master Mix 2× (Applied Biosystems) and Taqman (Applied Biosystems). 100 ng of each cDNA sample were used in the reaction as described in *SI Appendix*, *SI Materials and Methods*.

### Confocal Microscopy.

The sections were processed for indirect immunofluorescence. Antigen retrieval was performed using 0.01 M citrate buffer (pH 6.0) boiling in a microwave oven (1,300 W) twice for 5 min each. After washing, the slides were blocked with 3% donkey normal serum and 3% bovine serum albumin in PBS for 1 h at room temperature. The rabbit anti-Glut 2 (Santa Cruz Biotechnology, CA) primary antibody at 1:50 dilution was used. After incubation with the specific secondary antibody (Abcam, 1:100 dilution) donkey anti-rabbit (DyLight®594), the sections were washed and mounted in a commercial anti-fading agent with DAPI (Vectashield, Vector Laboratories, Burlingame, CA). Samples were examined and images captured by confocal laser scanning microscopy (Leica TCS SP5 II) in the Life Sciences Core Facility (LaCTAD) from the University of Campinas. No immunoreactivity was seen in control experiments in which primary antibodies were omitted.

### Glucose Tolerance Test.

The glucose tolerance tests were performed on mice that were fasted for 6 h. A fasting blood sample was taken to assess fasting glucose and insulin levels, and then the animals were challenged with an injection of 20% glucose into the peritoneum. The glucose and insulin levels were assessed in tail blood samples at 0, 15, 30, 45, 60, 90, and 120.

### PTT.

For the pyruvate tolerance test, mice fasted for 12 h followed by i.p. injection of sodium pyruvate solution (0.454 g/mL PBS - pH 7.4) at a dose of 1.5 g/kg body weight. Tail blood was taken at specified times, and glucose levels were measured by a glucometer (63).

### Glycerol Tolerance Test.

For the glycerol tolerance test, mice fasted for 12 h followed by i.p. injection of glycerol (0.333 g/mL PBS—pH 7.4) at a dose of 1.5 g/kg body weight. Tail blood was taken at specified times, and glucose levels were measured by a glucometer.

### FDG-PET/CT Imaging.

The animals fasted for 6 h before PET/CT scans (positron emission tomography/computed tomography). For a precise 18F-FDG injection and imaging acquisition, mice were anesthetized via intraperitoneal injection of Ketamine (100 mg/kg) and Xylazine (10 mg/kg). 37MBq (1 mCi) of 18F-FDG (in approximately 0.1 mL of NaCl 0.9% solution) was injected via caudal vein (*SI Appendix*, *SI Materials and Methods*).

### NMR.

#### 1H and 13C NMR spectroscopy-pyruvate injection.

1H-NMR spectra were acquired using a Varian Inova® spectrometer (Agilent Technologies Inc., Santa Clara, CA) equipped with a triple-resonance cold probe and operating at a 1H resonance frequency of 600 MHz. Spectra acquisition was performed with 256 scans collected with 32 K data points over a spectral width of 8,000 Hz (*SI Appendix*, *SI Materials and Methods*).

### Chromatography.

#### Chemicals and reagents.

All solvents and reagents used in this study were HPLC grade. Water was purified and deionized by the Milli-Q-UF system (Millipore, Milford, MA) and used throughout in all aqueous solutions. Acetonitrile, metformin hydrochloride 97%, ammonium acetate, ammonium hydroxide, Microcon YM-3 column (Amicon Ultra 0.5 mL) with a 3 kDa membrane filter, and 4-(dimethylamino)pyridine 99% (DMAP; internal standard) were purchased from Merck/Sigma-Aldrich (USA). The mobile phase used in the HPLC system was vacuum filtered through a 0.45 μm membrane (Sartorius Stedim Biotech, Göttingen, Germany).

### Serum and Tissue Samplings.

Different volumes of blood were collected from the cava and portal veins and left ventricle of the animals and immediately transferred to dry microtubes. These samples were centrifugated at 3,500 rpm for 15 min at room temperature. Afterward, the serum was collected and stored at −20 °C until assayed.

Tissue samples were obtained at different intervals after the administration of metformin solution orally. The entire heart, lungs, pancreas, spleen, gastrocnemius muscle, and left kidney were removed. Sections from the frontal lobe of the brain, the distal portion of the ileum, the colon, the liver, and the epididymal adipose tissue were also extracted. The samples were weighed and stored in sterile microtubes at −80 °C. For their processing, the microtubes were kept on ice. Metallic beads were added to the tissue, and the microtubes were subjected to agitation in the TissueLyser until all content was in suspension. The samples were stored at −80 ° C until the preparation for the HPLC analyses.

### HPLC Analysis.

According to *SI Appendix*, Table S3, all relevant parameters were set for the HPLC analysis and started the measurement. The chromatographic analysis was based on the quantification method reported by Labuzek et al. (64).

### Cell Culture.

The human colon cancer cell line Caco2 and human liver cancer cell line Huh7 were obtained from Dr. Marcelo Bispo de Jesus, from the Institute of Biology, UNICAMP. Caco2 cells were cultured in Dulbecco’s modified Eagle’s medium (DMEM) containing 20% FBS, and Huh7 cells were cultured in DMEM containing 10% FBS with the addition of antibiotics or fungicides. Both cell lines were maintained at 37 °C in a humid atmosphere and 5% CO2.

### Glucose Utilization and Glucose Production in Cells.

Glucose utilization in CaCo2 cells was calculated through glucose determination in the medium before and after metformin addition, in the following times: 1, 2, 4, 8, and 24 h. At 5 mM glucose concentration, glucose utilization was calculated by the differences between the times 8 and 4 h, and at 10 mM glucose, the differences between 24 and 4 h. These times were chosen after preliminary experiments that showed these times as the points when glucose concentration declined linearly. Glucose was determined by the glucose oxidase method.

Glucose production in Huh7 cells was calculated through the area over the basal glucose curve for 30 min after the addition of pyruvate to the cells. Samples from the medium were collected before and 5, 10, 15, 20, 25, and 30 min after pyruvate addition.

### Transfection.

A total of 2 × 10^4^ cells were seeded in a tissue culture plate in a complete growth medium and incubated overnight. On the day of transfection, 100 pmol of siRNA was diluted into OPTI-MEM (Life Technologies) and mixed with 10 μL of X-tremeGene siRNA Transfection Reagent (Roche) according to the supplier's protocol. The transfection medium was then replaced by a complete medium, and after 24 h, cells were treated with metformin (1 mmol/L) and incubated for an additional 24 h. Two siRNA for AMPK-PRKAA2 (EHU042081; Sigma-Aldrich) and PRKAA1 (EHU074041; Sigma-Aldrich), for Glut1—SLC2A1 (EHU028011; Sigma-Aldrich), for Glut2—SLC2A2 (EHU144201; Sigma-Aldrich), and for ATF4—ATF4 (EHU114901; Sigma-Aldrich) were used.

The animals were fasted for 6 h before siRNA administration. A total of 50 µg of siRNA Glut1—slc2a1 (EMU085671, Sigma-Aldrich) complexed with Invivofectamine® 3.0 reagent (Thermofisher) was administrated by oral gavage and intraperitoneally in mice 48 and 24 h before of treatment with metformin. Nontargeting control siRNA complexed with Invivofectamine® 3.0 Reagent was used at the same dose as a control.

Data are expressed as mean ± SD of the number of independent experiments indicated. The results of blots are presented as direct comparisons of bands in autoradiography and quantified by optical densitometry using the software ImageLab (v. 5.2.1 build 11, Bio-Rad © Laboratories). All data were analyzed for a normal distribution; normally distributed data were analyzed by two-tailed Student’s *t* test or one-way ANOVA, with the Bonferroni test for post hoc comparisons, when appropriate and non-normally distributed data were subjected to the Mann–Witney test. The level of significance adopted was *P* < 0.05 unless specified elsewhere. All graphs were made with GraphPad PRISM 7 (GraphPad, San Diego).

## Supplementary Material

Appendix 01 (PDF)

Dataset S01 (XLSX)

Dataset S02 (XLSX)

Dataset S03 (PPTX)

## Data Availability

All study data are included in the article and/or *SI Appendix*.
