# Supplementary material for: Metformin acts in the gut and induces gut-liver crosstalk
Source: Proc Natl Acad Sci U S A. 2023 Jan 19;120(4):e2211933120. doi: 10.1073/pnas.2211933120 (PMC9942892; doi:10.1073/pnas.2211933120)
Supplement: Supplementary file 1 — Appendix 01 (PDF) [file pnas.2211933120.sapp.pdf]

PNAS

Supporting Information for
Metformin acts in the gut and induces gut-liver crosstalk

Natália Tobar[1$], Guilherme Z. Rocha[1*$], Andrey Santos[1], Dioze Guadagnini[1], Heloísa B. Assalin[1], Juliana A. Camargo[1], Any E. S. S. Gonçalves[1], Flavia R. Pallis[1], Alexandre G. Oliveira[2], Silvana A. Rocco[3], Raphael M. Neto[3], Irene Layane de Sousa[3], Marcos R Alborghetti[3], Maurício L Sforça[3], Patrícia B. Rodrigues[4], Raissa G .Ludwig[5], Emerielle C. Vanzela[6], Sergio Q. Brunetto[7], Patrícia A. Boer[1] José A. R. Gontijo[1], Bruno Geloneze[1], Carla R. O. Carvalho[8], Patricia O. Prada[1,9], Franco Folli[1,13], Rui Curi[10], Marcelo A. Mori[5], Marco A R. Vinolo[4], Celso D. Ramos[11], Kleber G. Franchini[3], Claudio F. Tormena[12], Mario J. A. Saad[1*].

1 Department of Internal Medicine, School of Medical Sciences, University of Campinas, Campinas, Brazil
2 Department of Physical Education, São Paulo State University (UNESP), Rio Claro, Brazil
3 Brazilian Biosciences National Laboratory (LNBio) and Brazilian Center for Research in Energy and Materials (CNPEM), Campinas, Brazil.
4 Department of Genetics, Evolution, Microbiology and Immunology, Institute of Biology, University of Campinas, Campinas, Brazil
5 Department of Biochemistry and Tissue Biology, Institute of Biology, University of Campinas, Campinas, São Paulo, Brazil
6 Department of Structural and Functional Biology, University of Campinas
7 Biomedical Engineering Center, University of Campinas, Campinas, Brazil
8 Institute Biomedical Sciences, University of Sao Paulo-Department of Physiology and Biophysica
9 School of Applied Sciences, University of Campinas, Limeira, Brazil
10 Butantan Institute-São Paulo, Brazil
11 Division of Nuclear Medicine, Department of Radiology, School of Medical Sciences, University of Campinas, Campinas, Brazil.
12 Institute of Chemistry, University of Campinas.
13 Current address: Departmental Unit of Diabetes, Endocrinology and Metabolism, San Paolo Hospital, ASST Santi Paolo e Carlo; Department of Health Science, Università degli Studi di Milano, Milan, Italy.
$ These authors contributed equally to this work

Corresponding authors:
Mario J. A. Saad; e-mail: msaad@unicamp.br
Guilherme Z. Rocha e-mail: gzrocha@gmail.com

This PDF file includes:

Supporting text
Figures S1 to S7
Tables S1 to S3

Supporting Information Text

Materials and Methods.

1. **Real-time PCR**

Probe for animal tissues: GLUT1- Slc2a1 (Mm00441480_m1), GLUT2 - Slc2a2 (Mm00446229_m1) and ATF4-Atf4(Mm00515325_g). GAPDH (4352339E), b-actin-ACTB (4352341E) or B2M (Mm00437762_m1) expression were used as endogenous control, and samples from control mice were used as calibrators. 100 ng of each cDNA sample were used in the reaction with the following probe for Caco2 cells SLC2A1 (Hs00892681_m1) and ATF4 (Hs00900959_g1). GAPDH (4333764T), ACTB (4333762T) or B2M (HS00187842_m1) expression were used as endogenous control, and samples from control Caco2 cells were used as calibrators. A negative "No Template Control" was also included for each primer pair. Three replicas were run on the same plate for each sample. Results are expressed as relative expression values

2. **FDG-PET/CT imaging**

The radioactive activity was measured prior to and following injection to verify the net injected dose. Sixty minutes after 18F-FDG injection, each animal was subjected to PET/CT imaging in a prone position. The scans were performed in PET/CT imaging system (Siemens – Biograph mCT40). A CT image was acquired for attenuation correction purposes. CT acquisition conditions were set to 70 kV, 155 mA, and 0.5 mm slice thickness. The only bed was scanned for 15 minutes from head to tail. A 3-dimensional reconstruction model was used to analyze: OSEM 3D with 24 subsets and 2 interactions. PET and CT images were fused through True D software (Siemens). Regions of interest (ROI) were drawn by the semi-quantitative method (Isocontour), determining the maximal 18F-FDG uptake (SUVmax - standardized uptake value) in the intestine and in other tissues. SUV is defined as (A*W)/Ainj, where A (Bq/mL) is the radioactivity measured in a ROI, W (g) is the animal weight, and Ainj (Bq) is the activity of 18F-FDG injected. SUVmax is more accurate to estimate the true SUV than SUV mean for this kind of analysis.

3. **Nuclear Magnetic Resonance (NMR)**

1H and 13C NMR spectroscopy-pyruvate injection

A 1.5 s relaxation delay was incorporated between scans, during which a continual water pre-saturation radio frequency (RF) field was applied to eliminate residual water signal.

3.1 Sample preparation and spectra acquisition

Tissue samples were washed with cold saline (0.9% NaCl) and immediately ground to a powder under liquid nitrogen and kept in the -80°C freezer until the analysis by NMR. Powdered tissues samples were added to a cold methanol/chloroform solution (2:1 v/v, total of 0.5 mL) and sonicated (VCX 500, Vibra-Cell, Sonics & Material Inc., USA) for 3 min with a 10 s pause interval between each minute. A cold chloroform/deionized water solution (1:1 v/v, total of 0.5 mL) was then added to the samples. Samples were briefly vortexed and centrifuged at 3.1 × 103 g for 20 min at 4 °C. The upper phase was collected and dried in a vacuum concentrator (miVac Duo Concentrator, GeneVac, UK). The remaining solid phase was rehydrated in 0.6 mL of D2O-containing phosphate buffer (0.1 M, pH 7.4) and 0.5 mM of TMSPd4. Samples were added to a 5 mm NMR tube for immediate acquisition. 1H-NMR spectra were acquired using a Varian Inova® spectrometer (Agilent Technologies Inc., Santa Clara, CA, USA) equipped with a triple-resonance cold probe and operating at a 1H resonance frequency of 600 MHz. Spectra acquisition was performed with 256 scans collected with 32 K data points over a spectral width of 8000 Hz. A 1.5-s relaxation delay was incorporated between scans, during which a continual water pre-saturation radio frequency (RF) field was applied to eliminate residual water signal.

3.2 Quantification of metabolites

The metabolites were processed and quantified using NMR Suite software version 8.1 (Chenomx Inc™, Edmonton, AB, Canada). The processor module of this software was used to adjust the

spectral phase and baseline corrections. A 0.5 Hz line-broadening function was used to reduce signal noise and facilitate the fitting of the metabolite signals in spectral peaks. The water signal was suppressed, and the spectra were calibrated using the reference signal of the TMSP-d4 as 0.5 mM. The spectra were individually transferred to the Profiling module of this software to determine the metabolomic profile of each group. Metabolites were identified, and their concentrations were measured. Metabolite concentration data were exported to Excel® (Microsoft Office™ 365) and normalized when necessary.

A

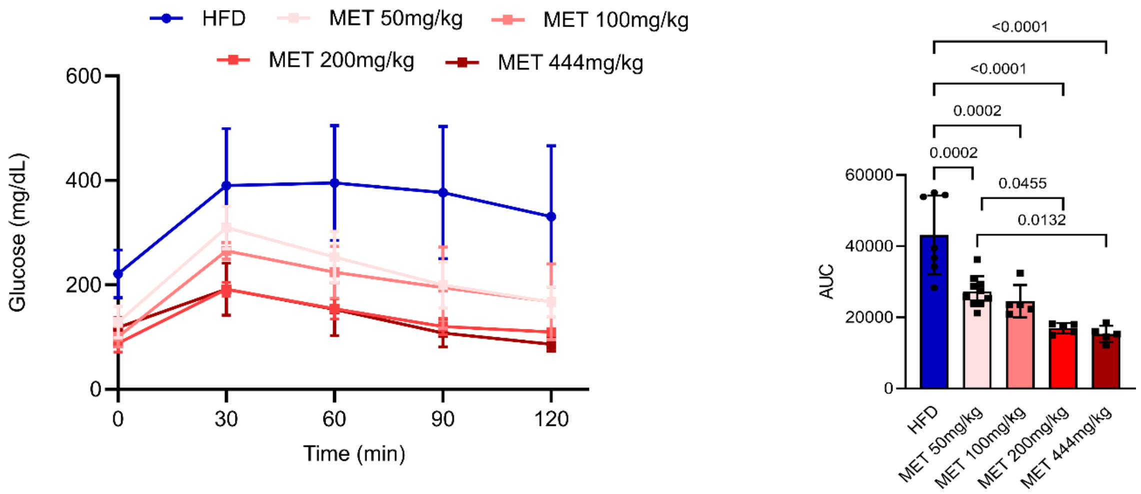

Fig. S1: A, Blood glucose levels from HFD and different doses of MET treated mice during a glucose tolerance test (GTT) and area under curve.

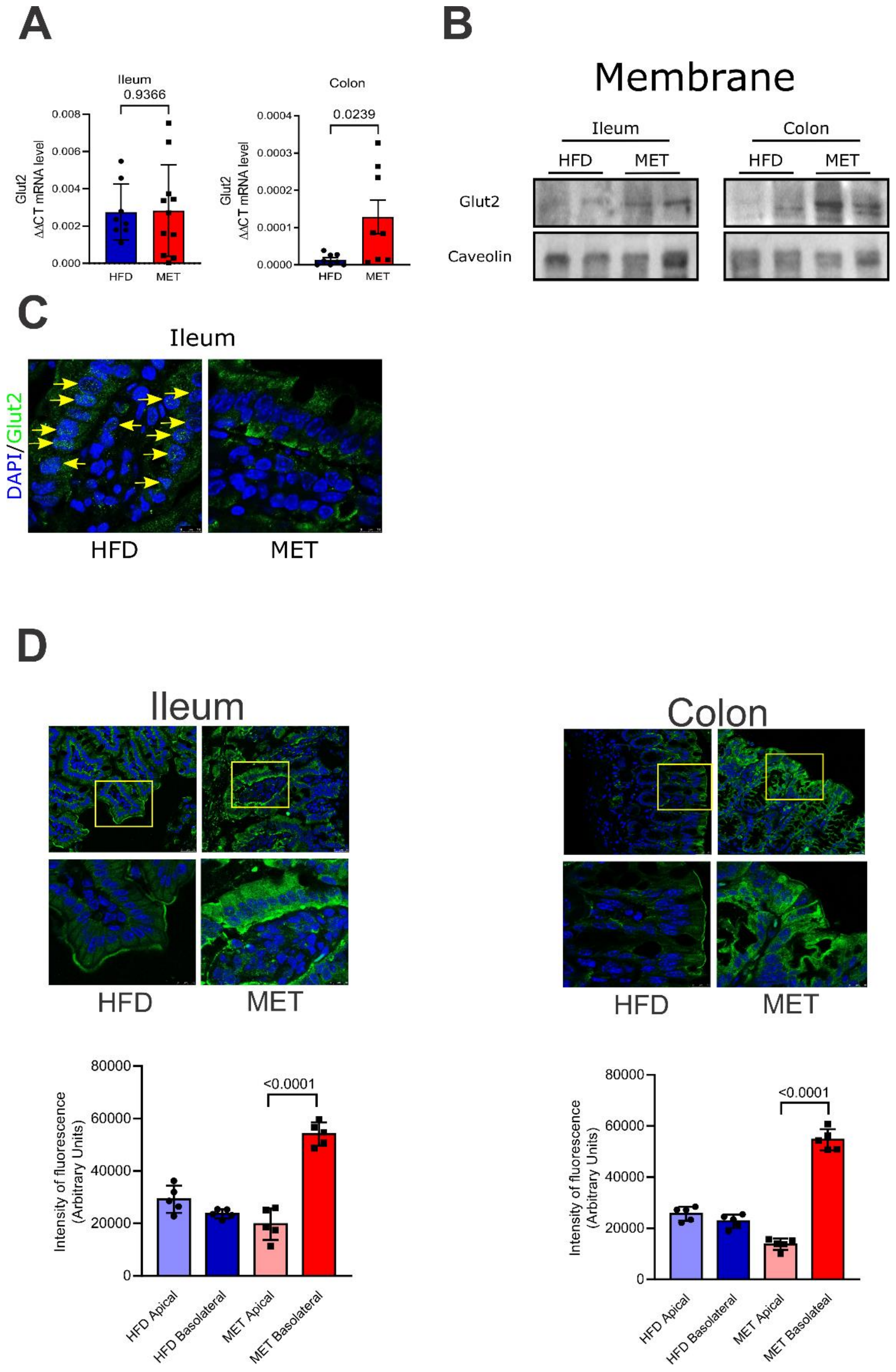

Fig. S2: A, Glut-2 mRNA expression in ileum and colon from HFD and MET mice (444 mg/kg for 10 days) **B,** Cytoplasmic membrane Glut-2 protein expression in ileum and colon from HFD and MET mice (444 mg/kg for 10 days). **C,** Confocal images of Glut-2 expression in ileum of HFD and MET mice (444 mg/kg for 10 days), arrows indicate nuclear localization of Glut-2. **D,** Confocal images of Glut-2 expression in the ileum and colon of HFD and MET mice and the corresponding bar graph indicated translocation of Glut-2 from the apical to the basolateral side after metformin.

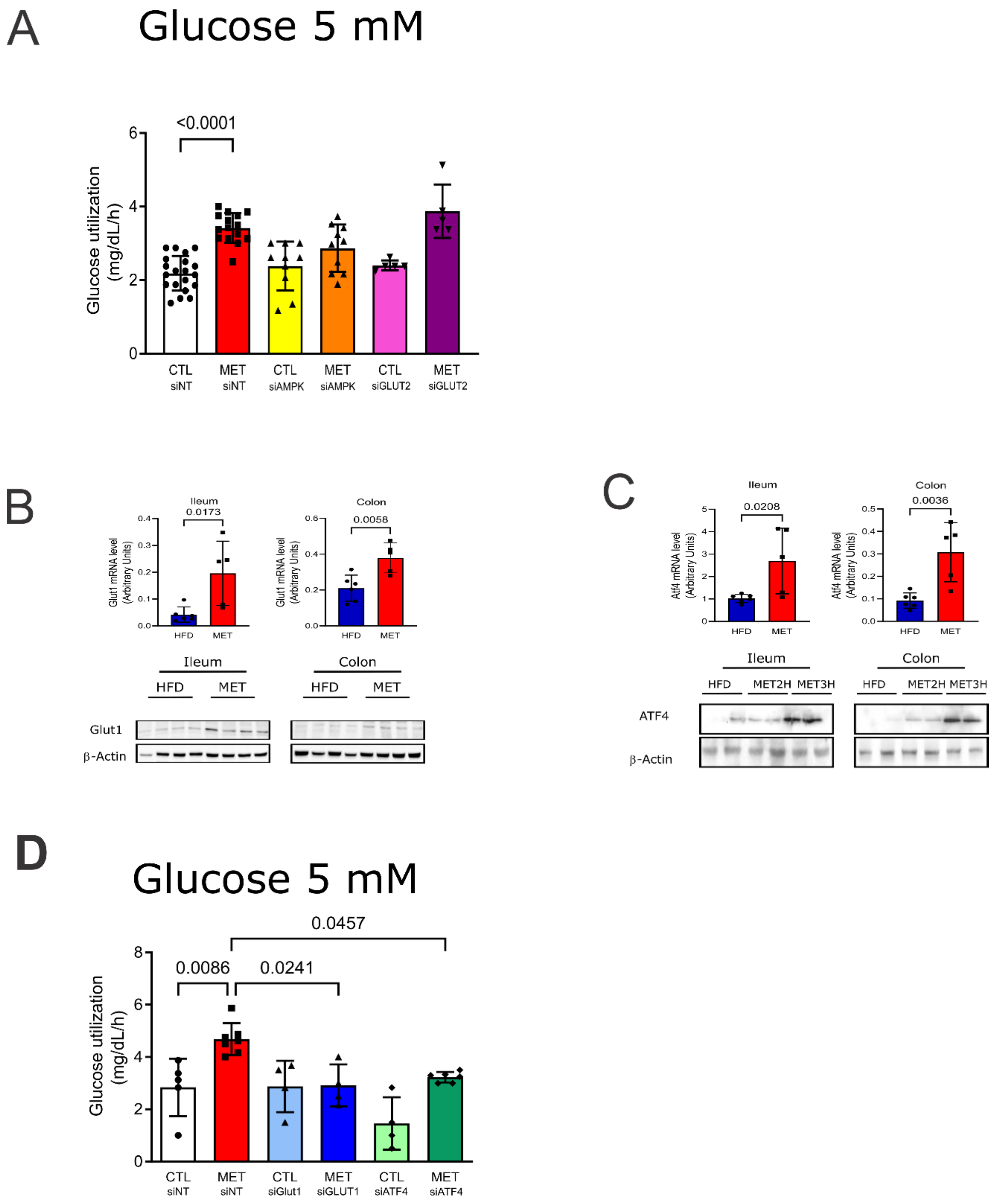

Fig. S3: A, Glucose uptake in physiological (5 mM) glucose concentrations in CaCo2 cells after the incubation with metformin (MET) in cells pre-treated with siRNA of AMPK and GLUT2 an non-targeting, as control (siNT) and protein expression showing the effect of the gene silencing. **B,** Glut-1 mRNA and protein expression in ileum and colon from HFD and MET mice (444 mg/kg for 10 days). **C,** ATF-4 mRNA expression in ileum and colon from HFD and MET mice (444 mg/kg for 10 days), and time course of metformin-induced (444 mg/kg) ATF4 protein expression in ileum and colon (n= 6). **D,** Glucose uptake in physiological (5 mM) glucose concentrations in CaCo2 cells after the incubation with metformin (MET) in cells pre-treated with siRNA of GLUT1 and ATF4 an non-targeting, as control (siNT).

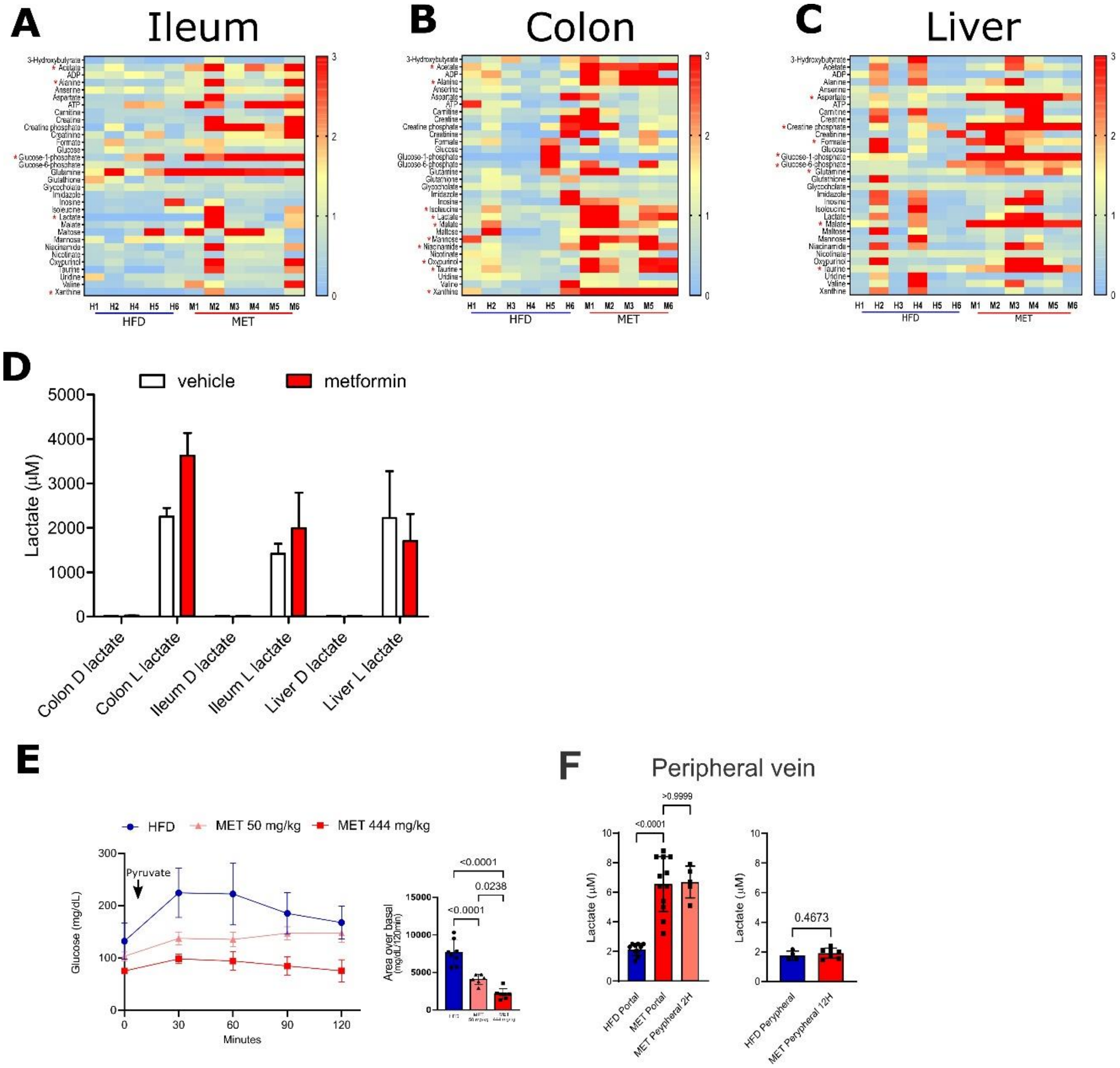

Fig S4: Metformin increases glucose anaerobic metabolism in ileum and colon and reduces hepatic glucose output in animals on HFD. A, B and C, Metabolomic analysis of ileum, colon, and liver from HFD and MET treated mice (444mg/kg for 10 days). **D,** RMN-spectroscopy analysis of lactate in ileum, colon and liver from the HFD and MET mice treated for 10 days (one-way ANOVA and Bonferroni's test, $p<0.05$, n=3) **E,** Pyruvate tolerance test curve and area over basal from HFD and MET(50 mg/kg and 444 mg/kg) treated mice (n=6)**. F,** Lactate in blood from portal and peripheral vein 2 h and 12 h after metformin treatment in Wistar rats.

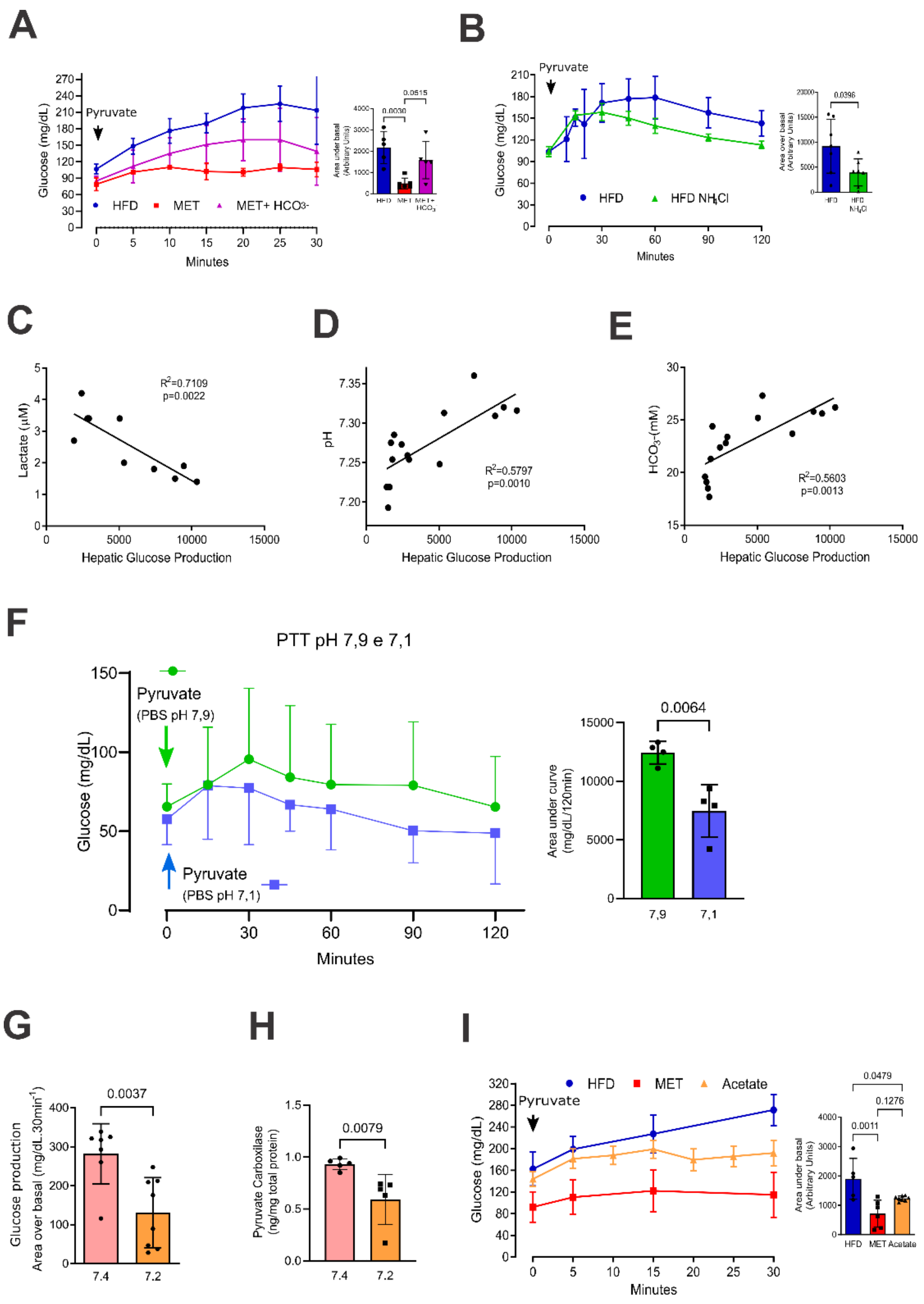

Fig. S5: **A,** Pyruvate tolerance test (30 min) curve and area over basal from Wistar rats on HFD, MET treated rats and MET (250 mg/kg) treated rats submitted to an ip injection of sodium bicarbonate (500 mg/kg) 30 min before pyruvate administration (n=5-6). **B,** Pyruvate tolerance test curve and area over basal of Wistar rats from HFD and HFD treated for 2 days by gavage with ammonium chloride (0.25M) (n=7). **C** Negative correlation between portal lactate levels and hepatic glucose production in obese rats treated or not with metformin (n=9). **D and E,** Positive correlation between pH or bicarbonate with hepatic glucose output in obese rats treated or not with metformin (n=15). **F,** Pyruvate tolerance test (120 min) curve and area over basal of control mice. The pyruvate (1,5g/kg) was diluted in PBS pH:7.9 or pH:7.1. **G,** reduced glucose production after a decrease in pH from 7.4 to 7.2. **H,** pyruvate carboxylase protein expression in HuH7 cells in different pH. **I,** Pyruvate tolerance test (30 min) curve and area over basal from Wistar rats on HFD, MET and MET submitted to an i.p. injection of sodium acetate (50 mg/kg) (n=5-8).

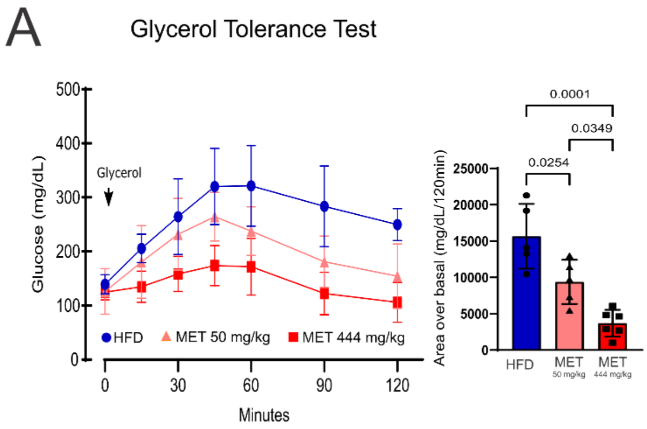

Fig. S6: **A,** Glycerol tolerance test curve and area under curve of mice treated with metformin at low (50 mg/kg) and high dose (444 mg/kg) (n=5).

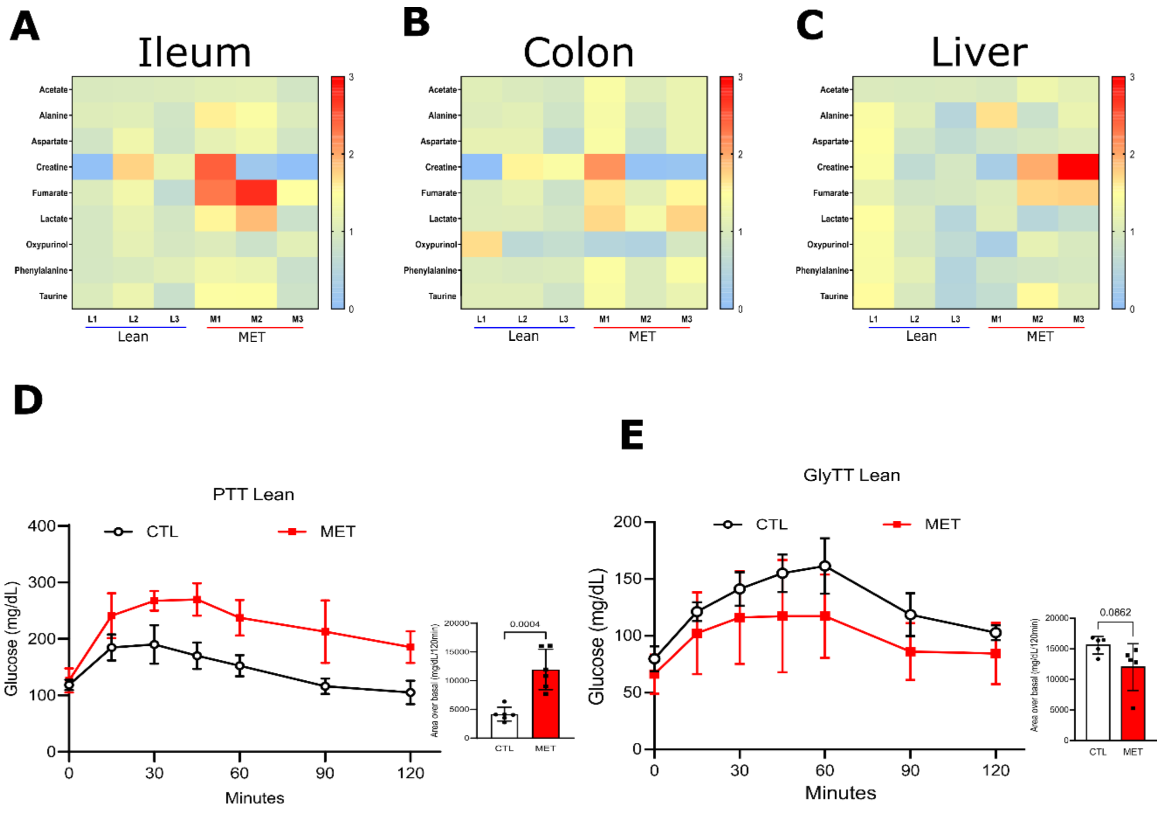

Fig. S7: A, B and C, Metabolomic analysis of ileum, colon and liver from lean mice treated with vehicle or and MET (50 mg/kg for 10 days) (n=3). **D,** Pyruvate **and E,** Glycerol tolerance tests curve and area under curve of metformin treated lean mice at low dose 50 mg/kg) (n=5).

Table S1. Baseline Features of Study Participants by Treatment Group

	CTL	DM	DM+MET	p-value
**Sex (M/F)**	8/3	2/3	9/7	0.2517
**Age (years)**	52	62	58	CTL vs DM 0.6195<br>CTL vs DM+MET 0.8957<br>D vs D MET >0.9999
**BMI (kg/m^2)**	22.3	25.7	27.3	CTL vs DM 0.3025<br>**CTL vs DM+MET 0.0115**<br>D vs D MET 0.6969
**Gucose (mg/dL)**	89	112	125	CTL vs DM 0.5618<br>**CTL vs DM+MET 0.0027**<br>DM vs DM+ MET 0.1988
**Antihypertensive treatment (%)**	60%	60%	75%	0.2390

Table S2 Metformin concentration in portal vein and tissues after treatment with 50 mg/kg or 444 mg/kg for ten days in HFD mice. Samples from portal vein, liver and ileum were collected two hours after and colon eight hours after last gavage.

	Metformin		
**Tissue**	**50 mg/kg**	**444 mg/kg**	**p-value**
**Portal vein (µmol/L)**	10,4 ± 4,2	25,1 ± 3,9	0,000434
**Liver (µmol/kg wet weight)**	157,2 ± 63,8	317,3 ± 114,4	0,02572
**Ileum (µmol/kg wet weight)**	539,9 ± 257,7	814,8 ± 514,9	0,317006
**Colon (µmol/kg wet weight)**	824,5 ± 178,5	1013,9 ± 142,2	0,100479

Table S3. Conditions and parameters to be used for HPLC-UV analyses of metformin in plasma and tissues matrices.

HPLC system	Waters Alliance 2695 (Milford, MA, USA), equipped with a quaternary pump, sample manager, and degasser	
Detector	Waters 2996 Uv-Vis set in 210-400 nm range	
System control, data acquisition, and processing	Waters Empower 2002 chromatography software	
Column	XSelect CSH™ Fluoro-Phenyl (150 x 4.6 mm I.D.; 3.5 ìm particle size) Waters	
Guard Column	XSelect CSH™ Fluoro-Phenyl (20 x 4.6 mm I.D.; 3.5 ìm particle size) Waters	
Mobile phase	Solvent A- Acetonitrile Solvent B- 0.010 M ammonium acetate, pH 7.6	
Isocratic conditions	Time (min.)	8
	A (%)	47.8
	B(%)	52.2
	Flow	1.0 mL/min
	Injection volume	25 ìL
	Temperature	30°C
	Metformin and DMAP (IS) Detections	UV @ 233 and 280 nm
	Run time	8 minutes
