# Supplementary material for: Metformin acts in the gut and induces gut-liver crosstalk
Source: Proc Natl Acad Sci U S A. 2023 Jan 19;120(4):e2211933120. doi: 10.1073/pnas.2211933120 (PMC9942892; doi:10.1073/pnas.2211933120)
Supplement: Supplementary file 4 — Dataset S03 (PPTX) [file pnas.2211933120.sd03.pptx]

## Slide 1
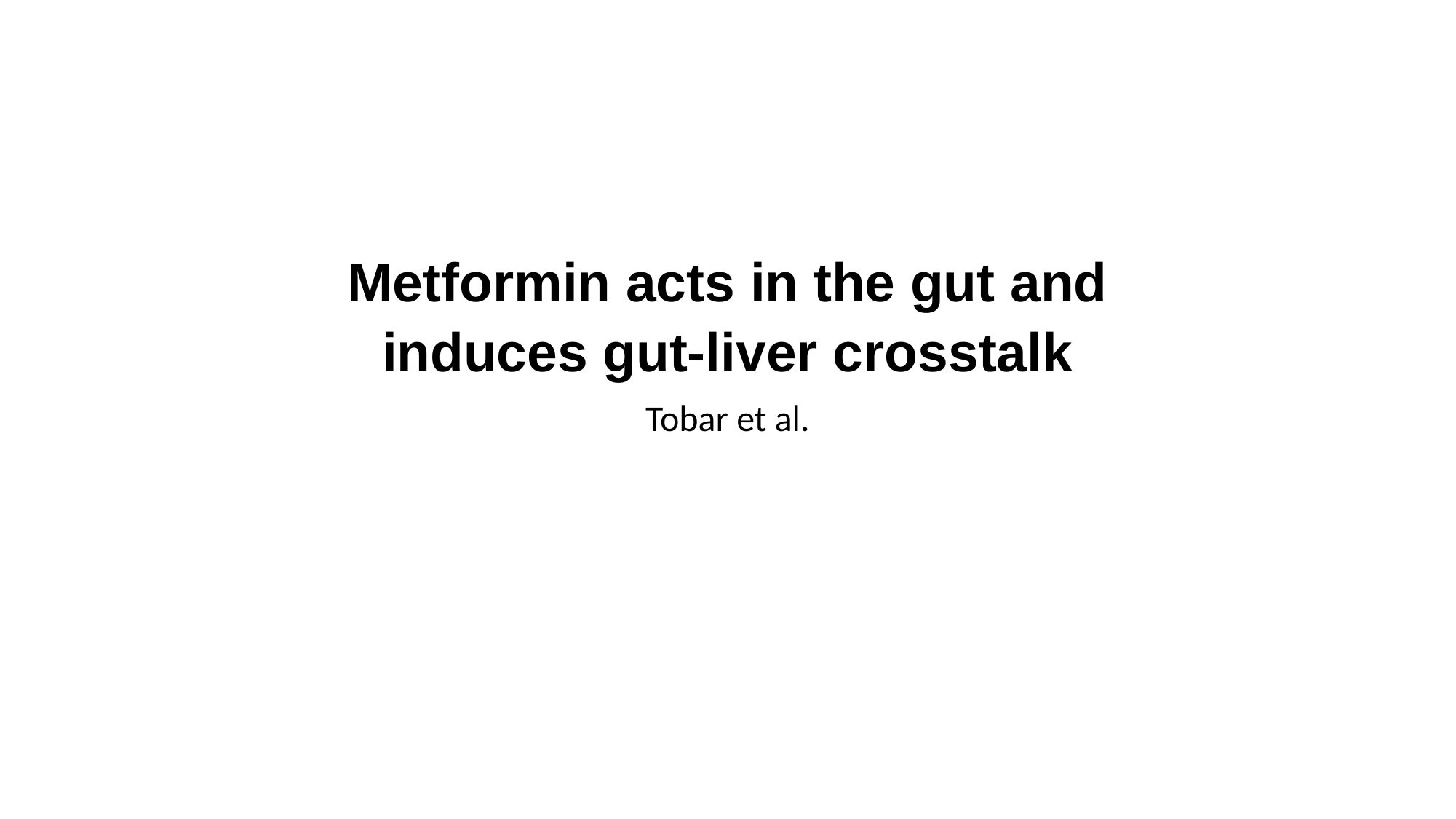

Metformin acts in the gut and induces gut-liver crosstalk
Tobar et al.

## Slide 2
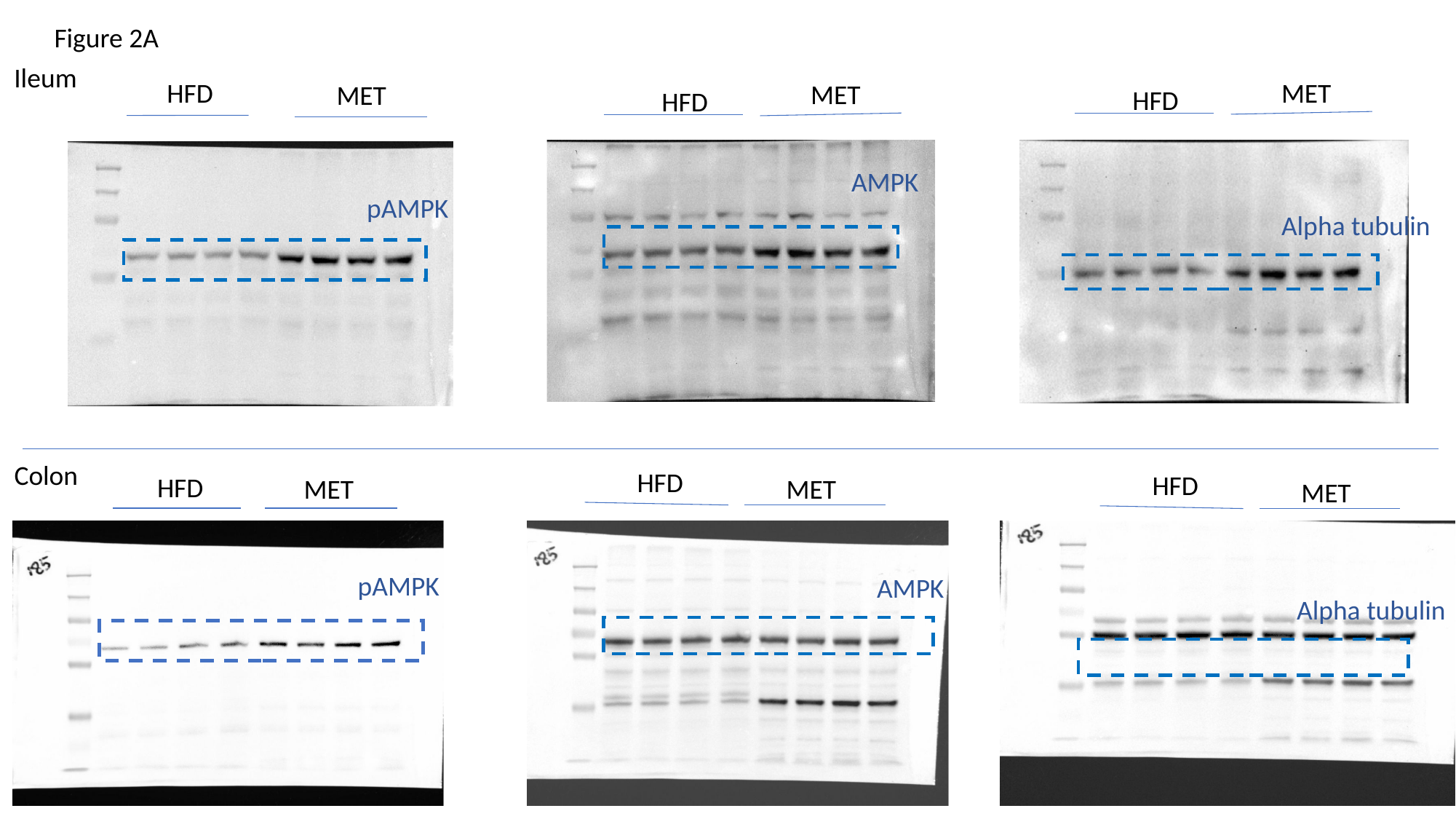

Figure 2A
Ileum
MET
HFD
MET
MET
HFD
HFD
AMPK
pAMPK
Alpha tubulin
Colon
HFD
HFD
HFD
MET
MET
MET
pAMPK
AMPK
Alpha tubulin

## Slide 3
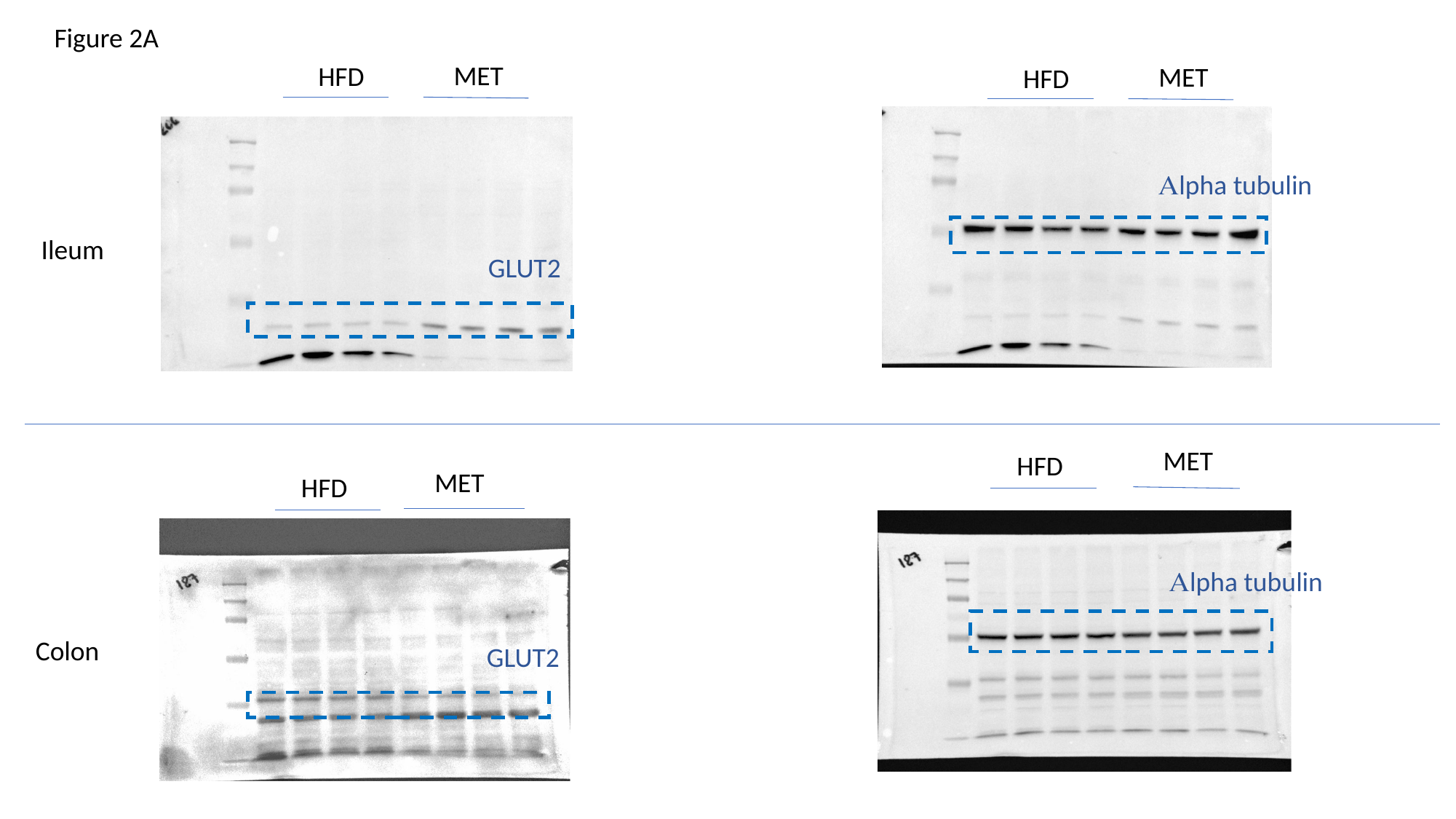

Figure 2A
MET
HFD
MET
HFD
Alpha tubulin
Ileum
GLUT2
MET
HFD
MET
HFD
Alpha tubulin
Colon
GLUT2

## Slide 4
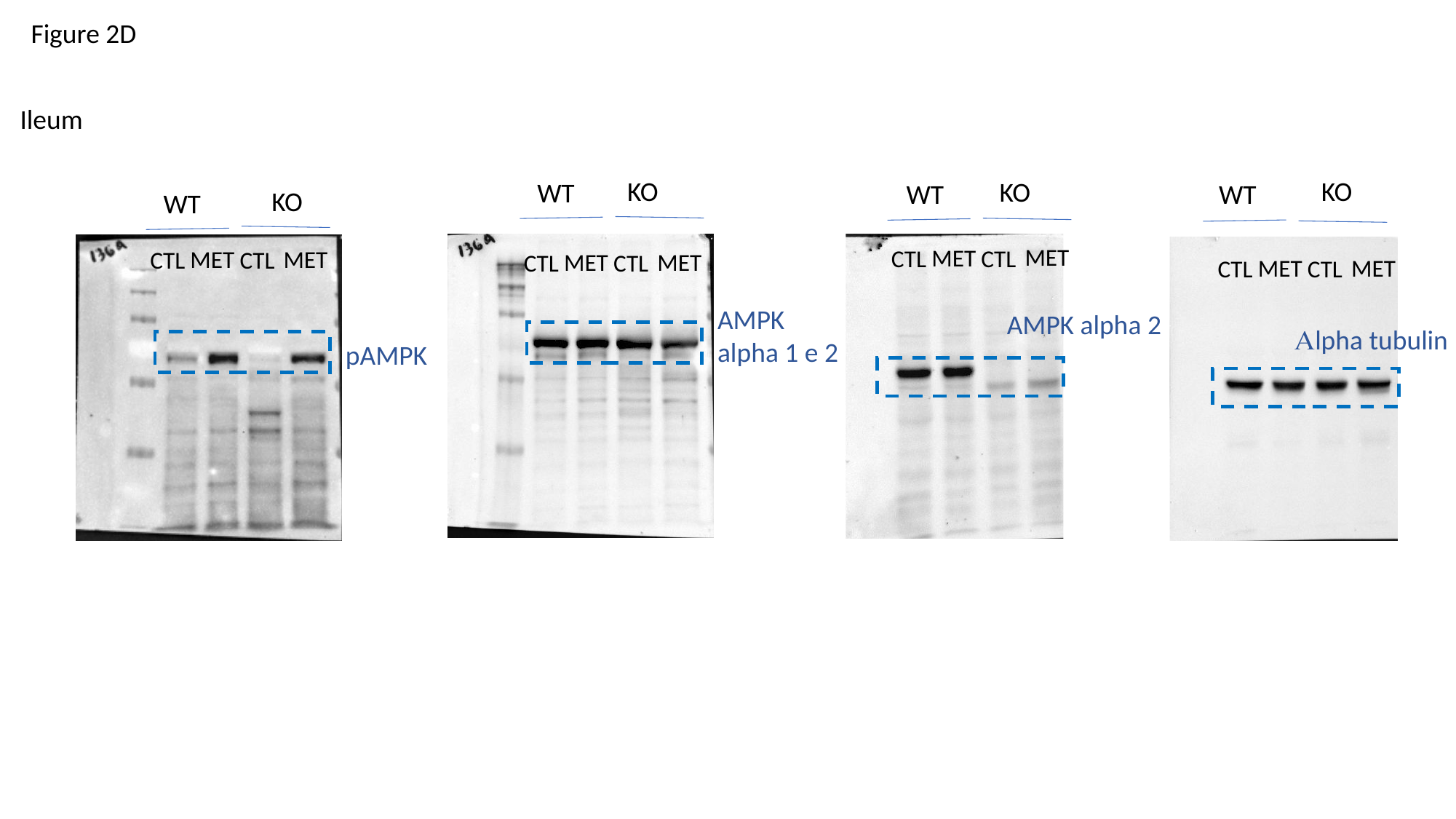

Figure 2D
Ileum
KO
KO
KO
WT
WT
WT
KO
WT
MET
MET
CTL
CTL
MET
MET
CTL
CTL
MET
MET
CTL
CTL
MET
MET
CTL
CTL
AMPK alpha 1 e 2
AMPK alpha 2
Alpha tubulin
pAMPK

## Slide 5
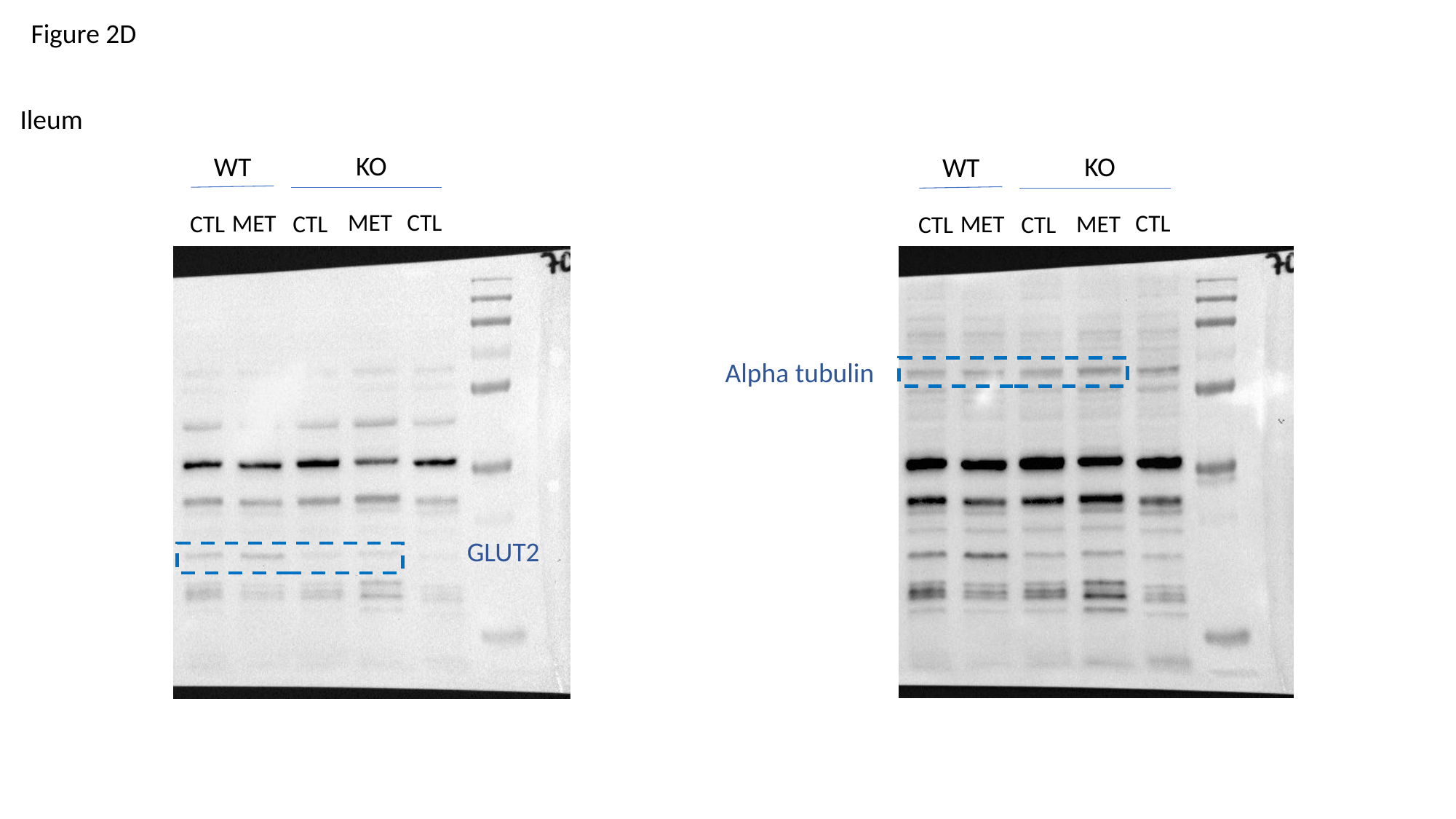

Figure 2D
Ileum
KO
KO
WT
WT
CTL
MET
MET
CTL
MET
CTL
MET
CTL
CTL
CTL
Alpha tubulin
GLUT2

## Slide 6
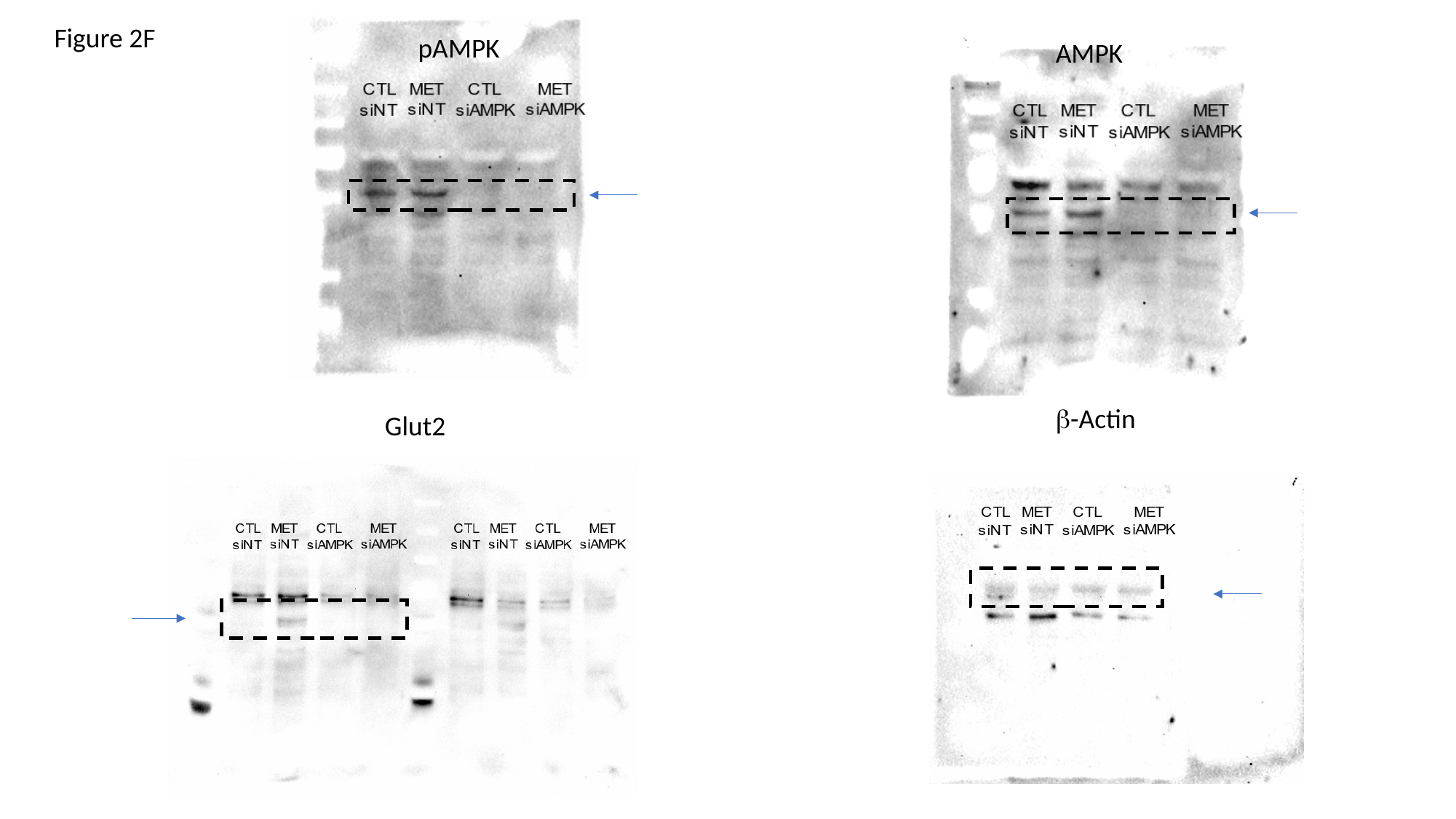

Figure 2F
pAMPK
AMPK
b-Actin
Glut2

## Slide 7
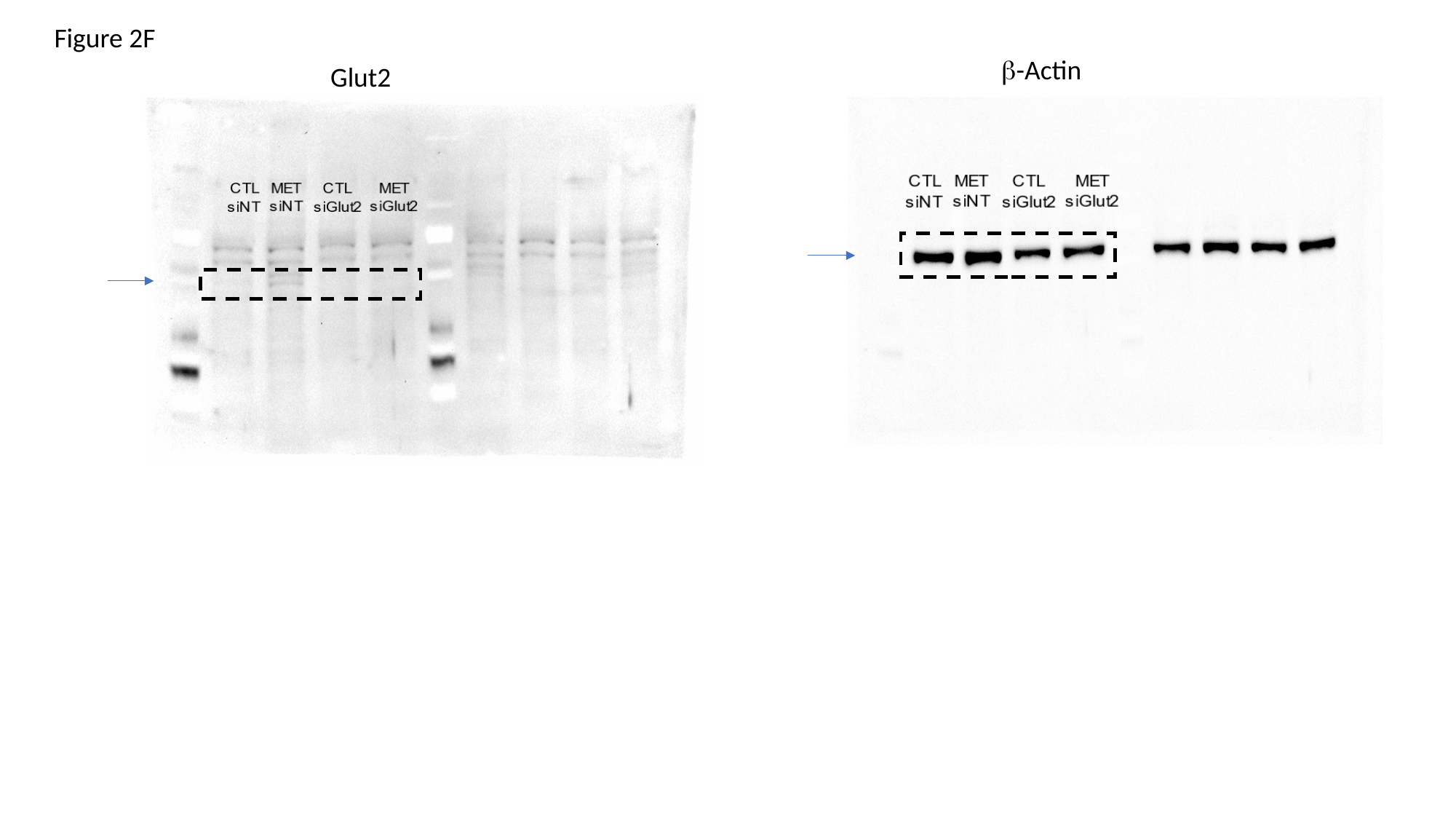

Figure 2F
b-Actin
Glut2

## Slide 8
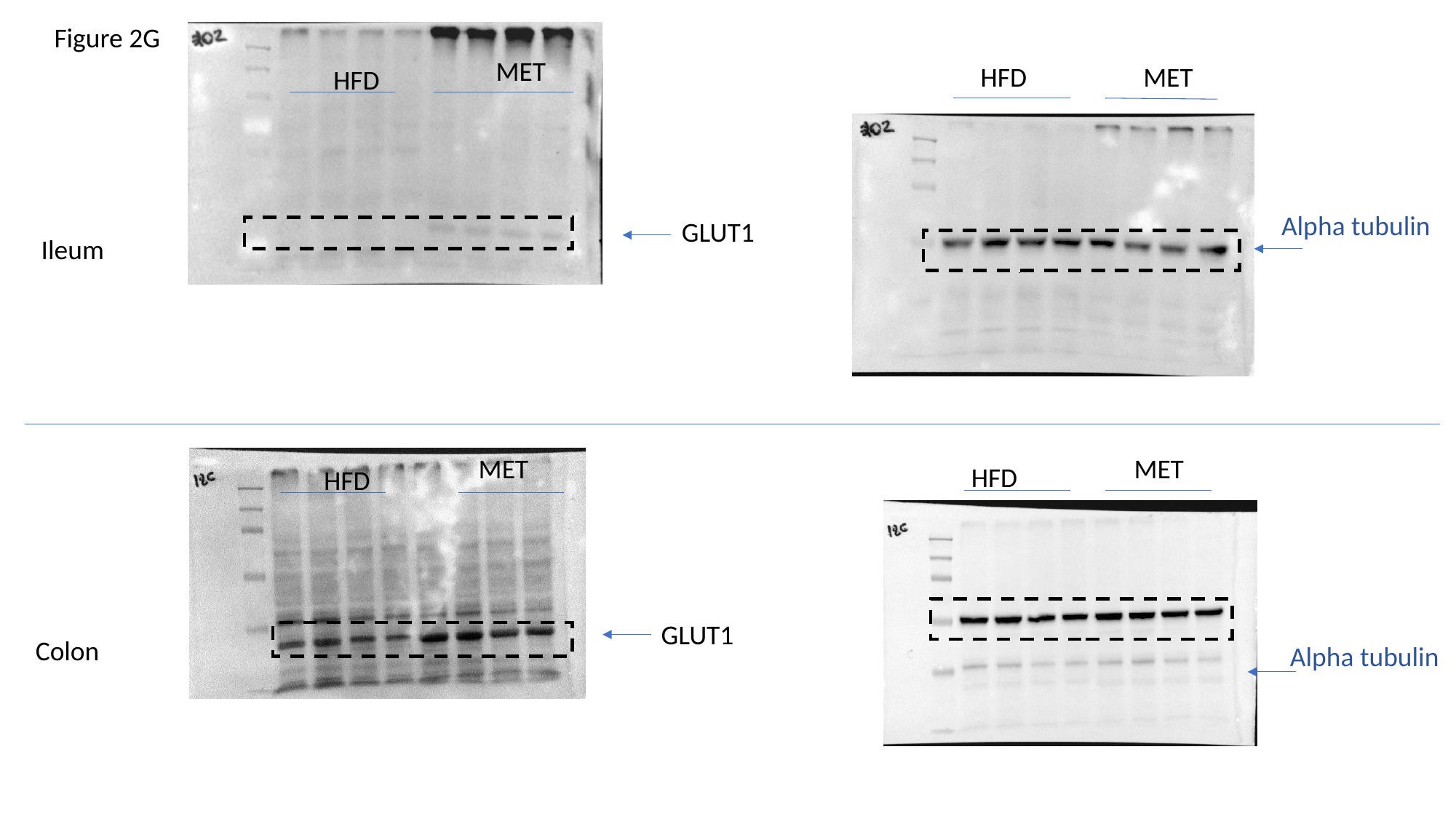

Figure 2G
MET
HFD
MET
HFD
Alpha tubulin
GLUT1
Ileum
MET
MET
HFD
HFD
GLUT1
Colon
Alpha tubulin

## Slide 9
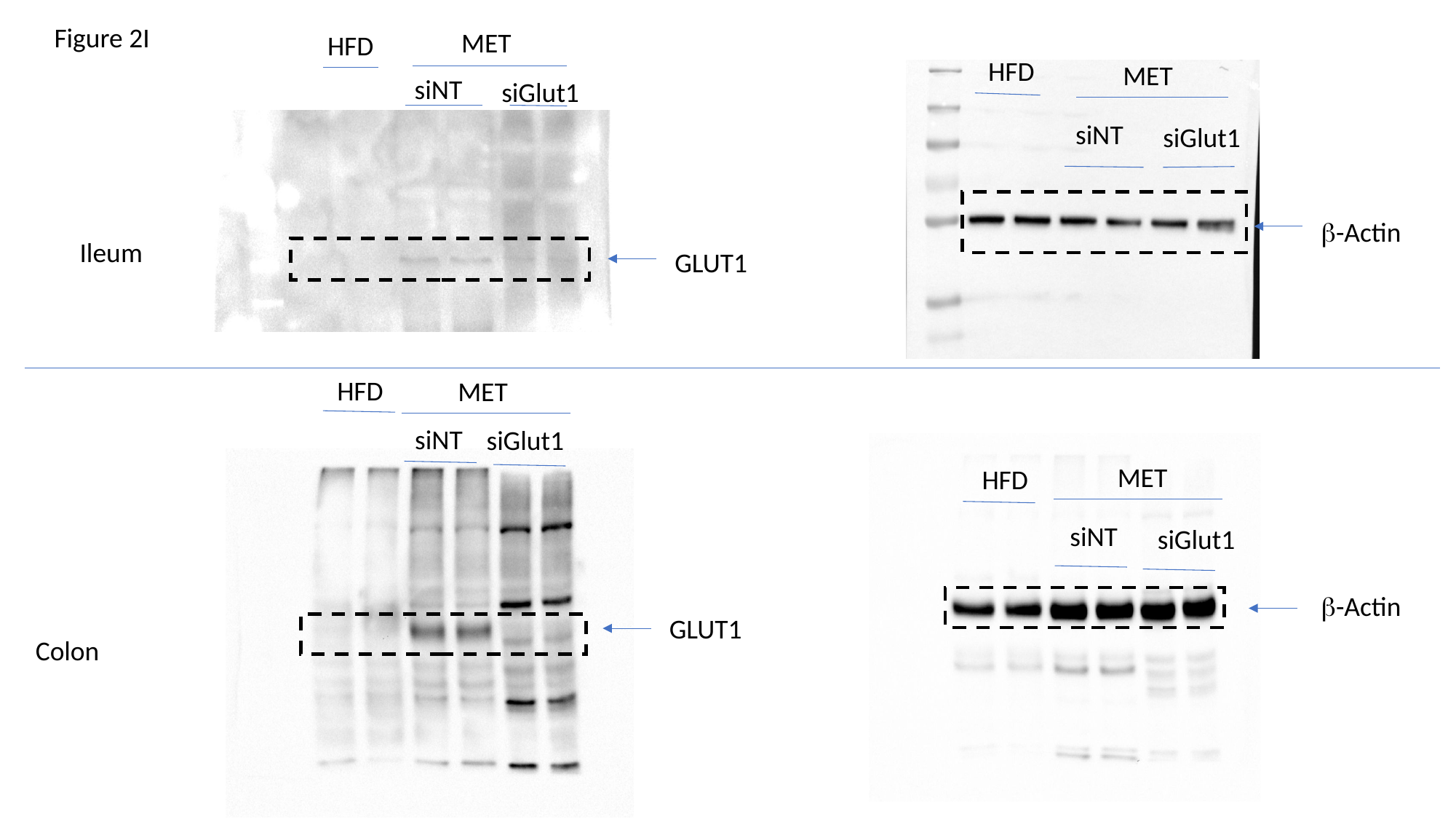

Figure 2I
MET
HFD
HFD
MET
siNT
siGlut1
siNT
siGlut1
b-Actin
Ileum
GLUT1
HFD
MET
siNT
siGlut1
MET
HFD
siNT
siGlut1
b-Actin
GLUT1
Colon

## Slide 10
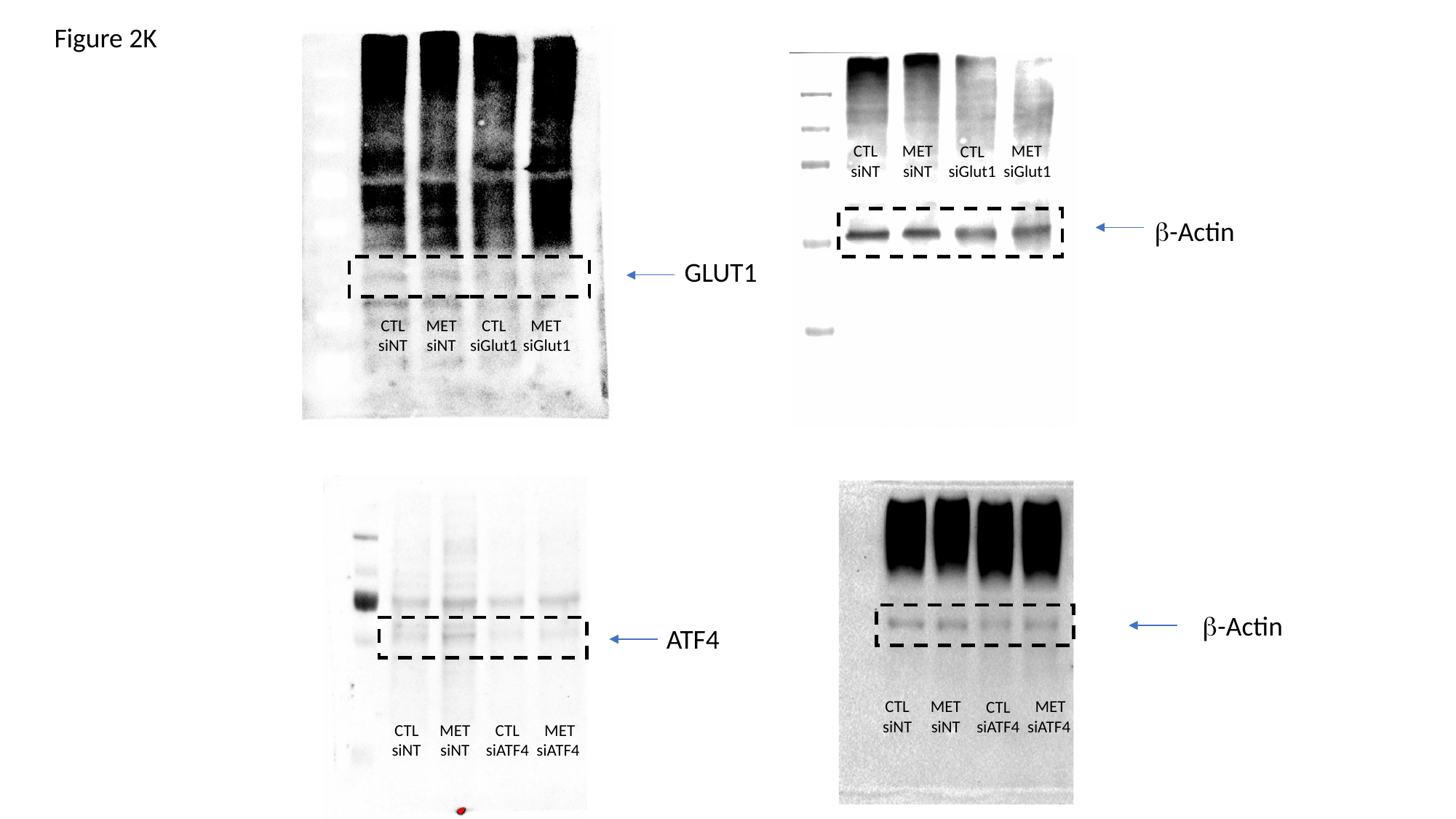

Figure 2K
CTL
siNT
MET
siNT
 MET
siGlut1
CTL
siGlut1
b-Actin
GLUT1
CTL
siNT
MET
siNT
 MET
siGlut1
CTL
siGlut1
b-Actin
ATF4
CTL
siNT
MET
siNT
 MET
siATF4
CTL
siATF4
CTL
siNT
MET
siNT
 MET
siATF4
CTL
siATF4

## Slide 11
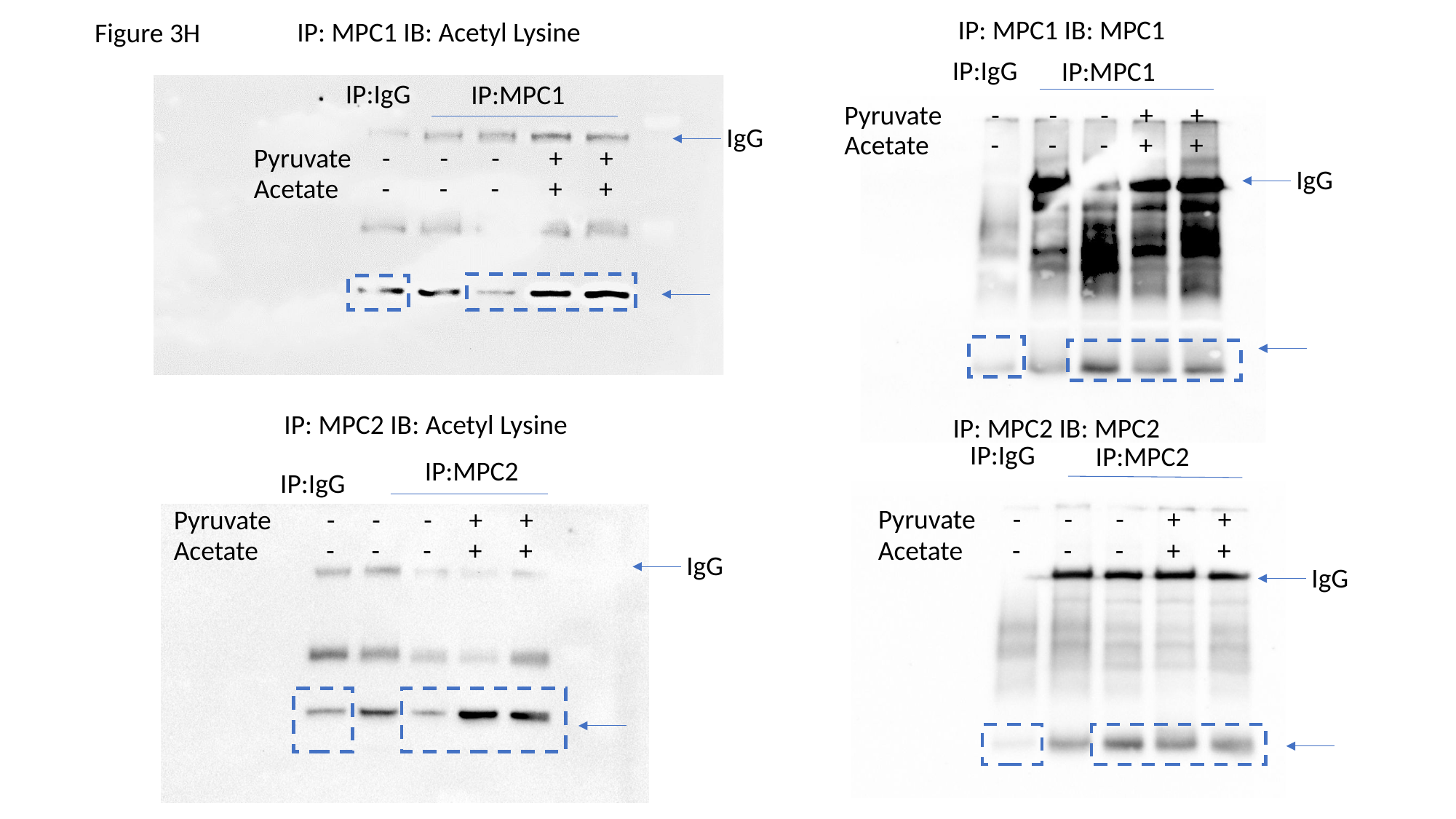

IP: MPC1 IB: MPC1
IP: MPC1 IB: Acetyl Lysine
Figure 3H
IP:IgG
IP:MPC1
IP:IgG
IP:MPC1
Pyruvate - - - + +
IgG
Acetate - - - + +
Pyruvate - - - + +
IgG
Acetate - - - + +
IP: MPC2 IB: Acetyl Lysine
IP: MPC2 IB: MPC2
IP:IgG
IP:MPC2
IP:MPC2
IP:IgG
Pyruvate - - - + +
Pyruvate - - - + +
Acetate - - - + +
Acetate - - - + +
IgG
IgG

## Slide 12
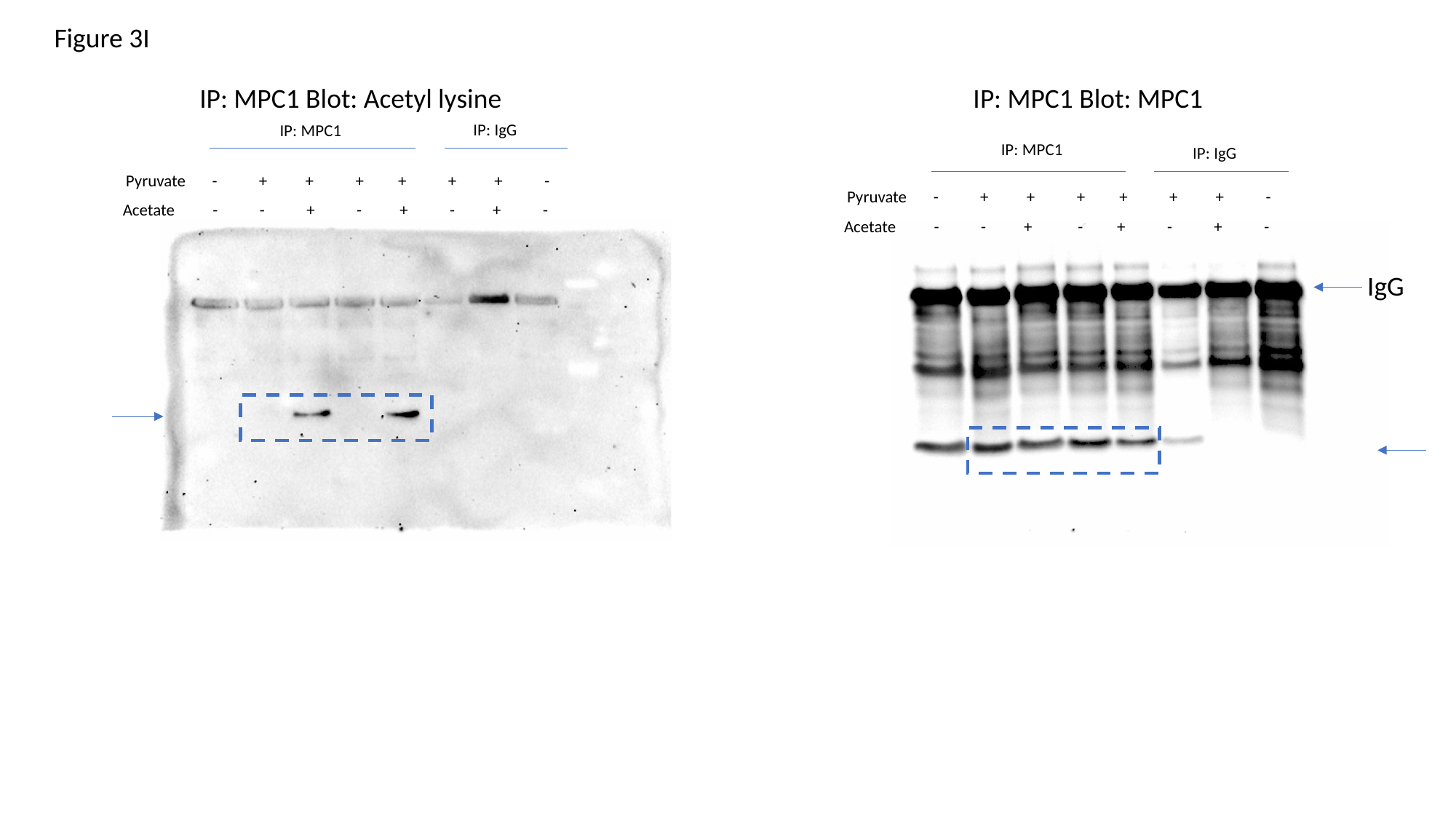

Figure 3I
IP: MPC1 Blot: Acetyl lysine
IP: MPC1 Blot: MPC1
IP: IgG
IP: MPC1
IP: MPC1
IP: IgG
Pyruvate - + + + + + + -
Pyruvate - + + + + + + -
Acetate - - + - + - + -
Acetate - - + - + - + -
IgG

## Slide 13
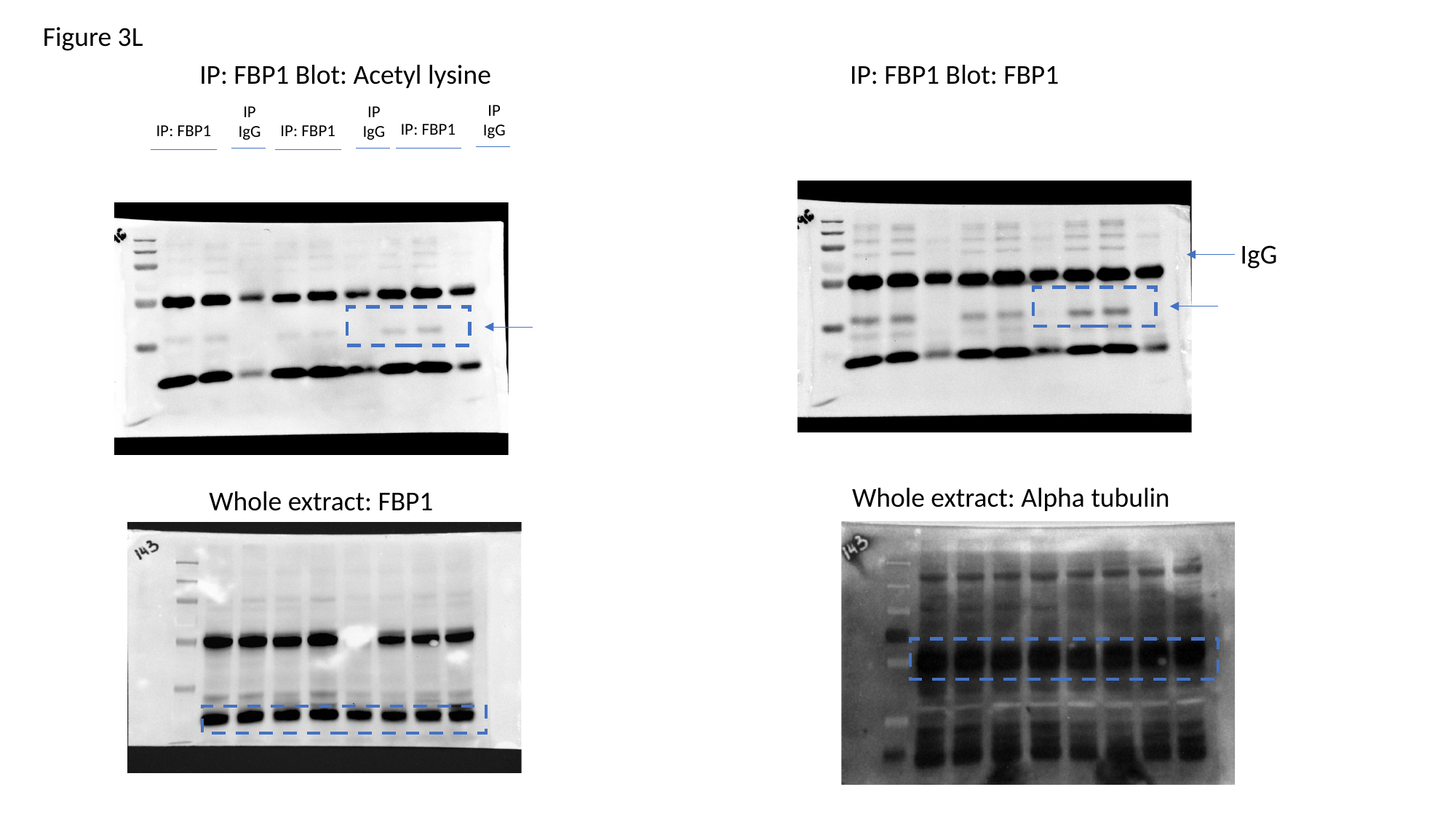

Figure 3L
IP: FBP1 Blot: Acetyl lysine
IP: FBP1 Blot: FBP1
IP IgG
IP IgG
IP IgG
IP: FBP1
IP: FBP1
IP: FBP1
IgG
Whole extract: Alpha tubulin
Whole extract: FBP1

## Slide 14
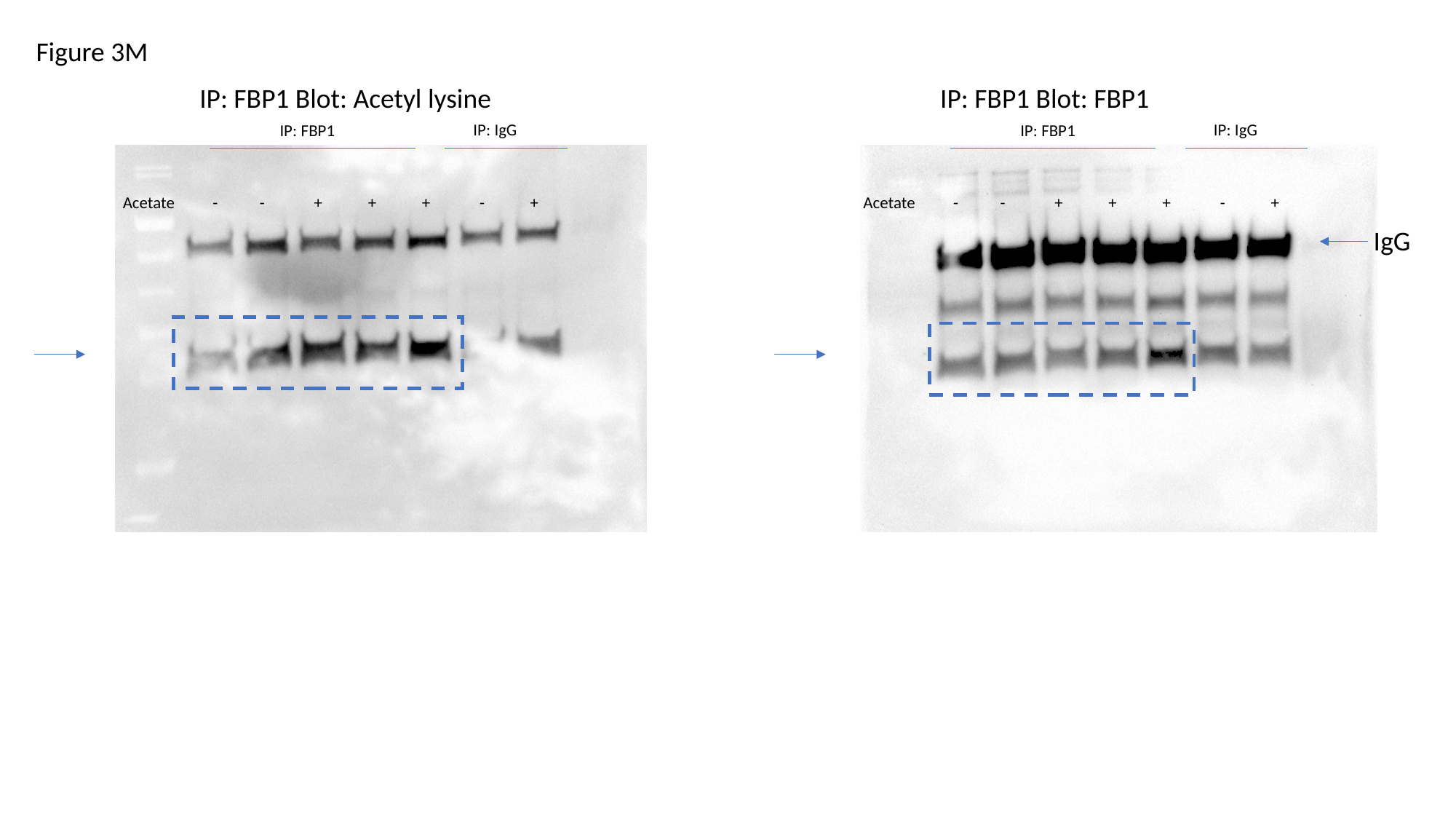

Figure 3M
IP: FBP1 Blot: Acetyl lysine
IP: FBP1 Blot: FBP1
IP: IgG
IP: IgG
IP: FBP1
IP: FBP1
Acetate - - + + + - +
Acetate - - + + + - +
IgG

## Slide 15
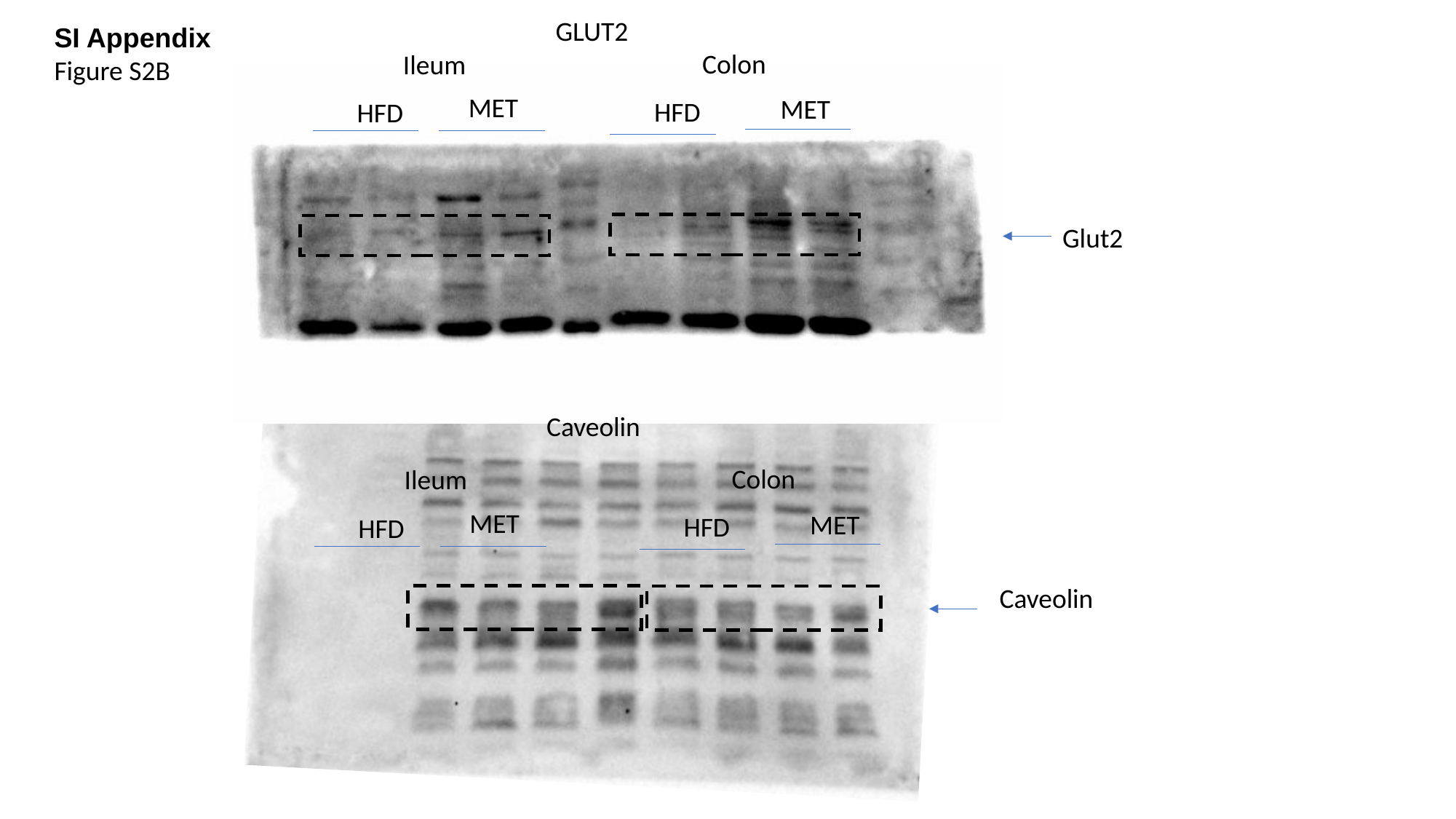

GLUT2
SI Appendix Figure S2B
Colon
Ileum
MET
MET
HFD
HFD
Glut2
Caveolin
Colon
Ileum
MET
MET
HFD
HFD
Caveolin

## Slide 16
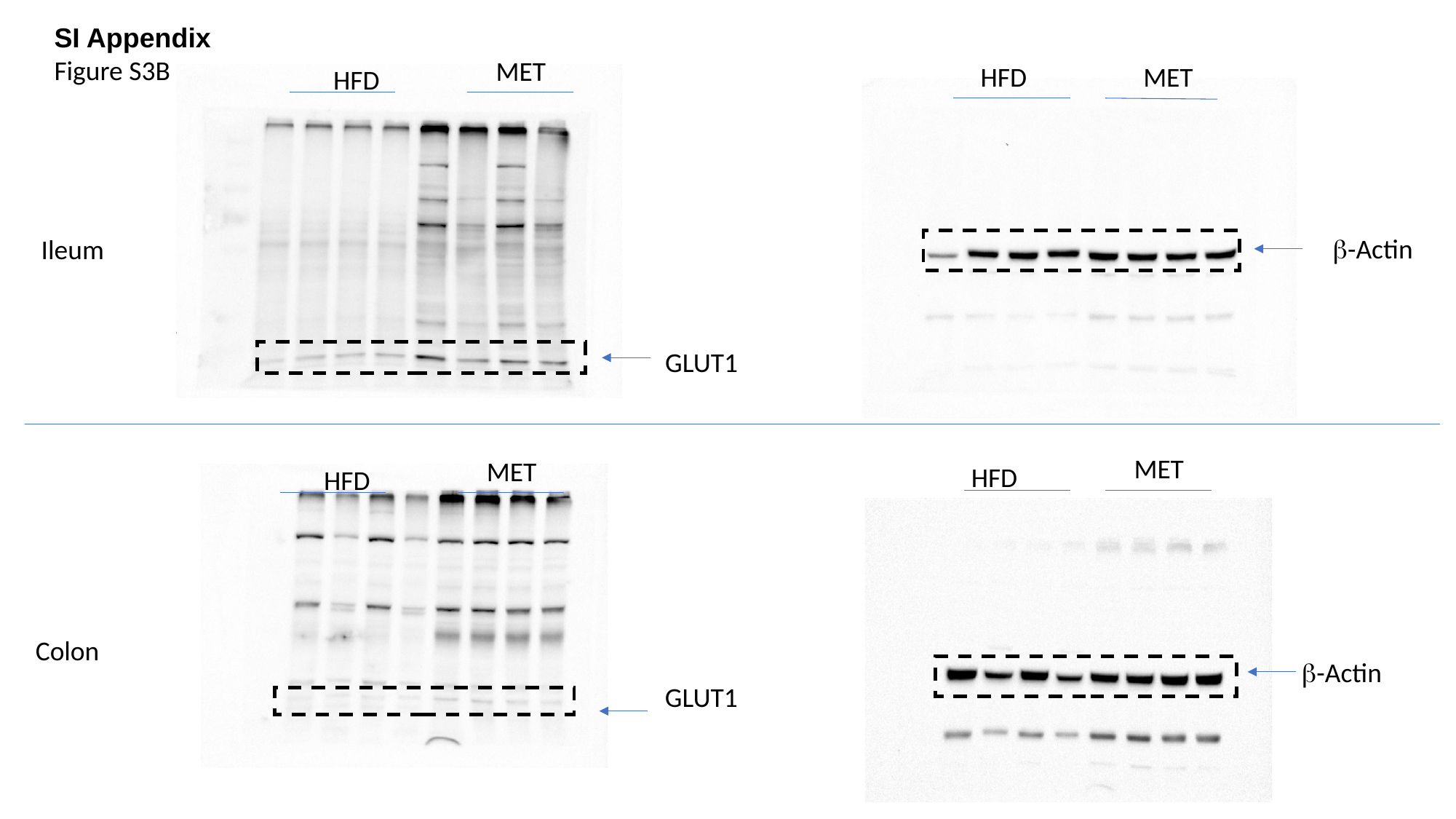

SI Appendix Figure S3B
MET
HFD
MET
HFD
b-Actin
Ileum
GLUT1
MET
MET
HFD
HFD
Colon
b-Actin
GLUT1

## Slide 17
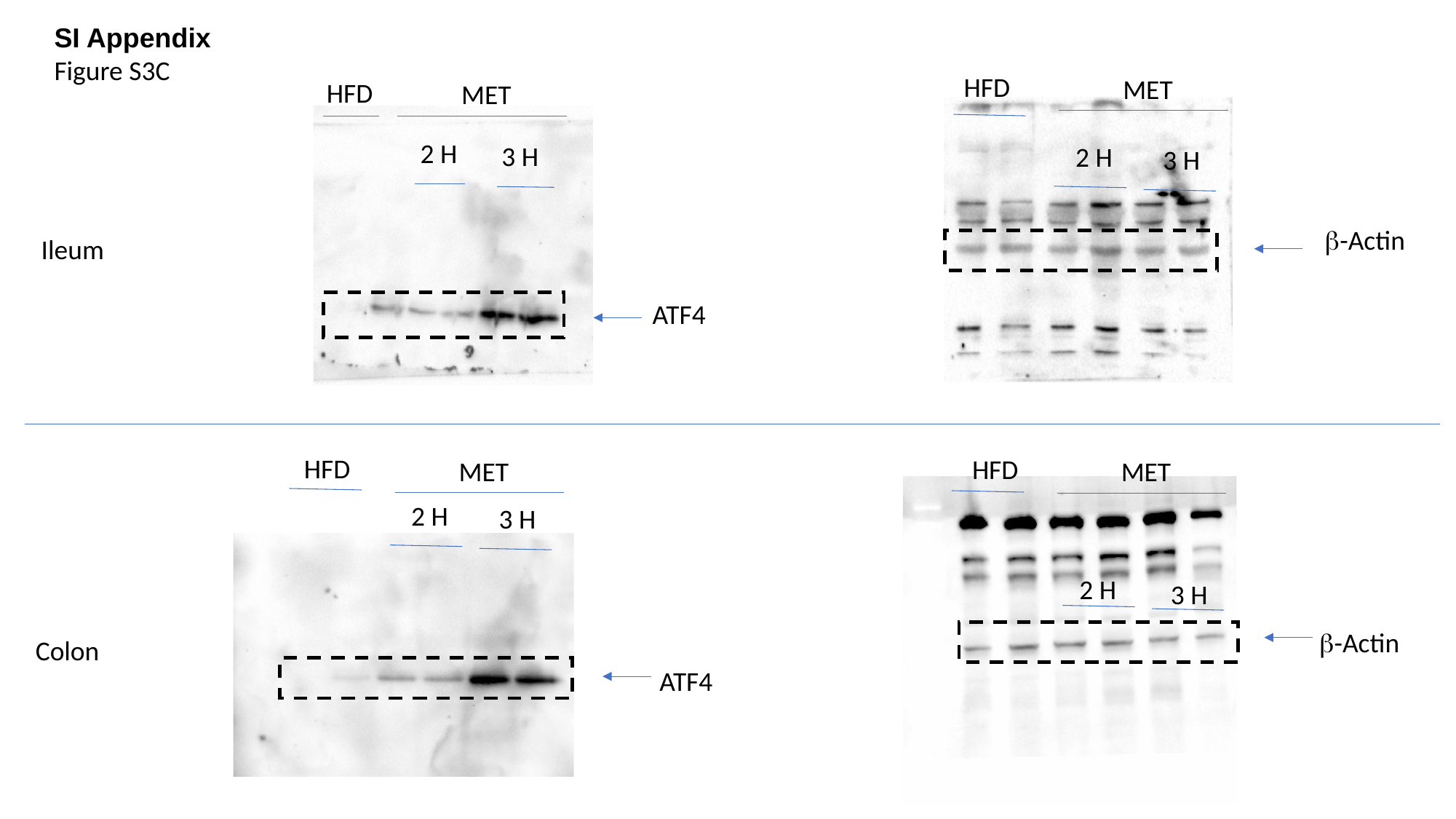

SI Appendix Figure S3C
HFD
MET
HFD
MET
2 H
3 H
2 H
3 H
b-Actin
Ileum
ATF4
HFD
HFD
MET
MET
2 H
3 H
2 H
3 H
b-Actin
Colon
ATF4
